# Verbal de-escalation techniques for the prevention and management of workplace violence in healthcare: a systematic review

**DOI:** 10.3389/fpsyt.2026.1819502

**Published:** 2026-10-14

**Authors:** Hajar Seiyad, Joseph Nimako, Brendan Singh, Brendan Lyver, Stephen Villavicencio, Melanie Anderson, Robert Whiteside, John Shannon, Alize Chandler, Torie Vaughan, Marc Toppings, Rima Styra, Christian Schulz-Quach

**Affiliations:** 1 University Health Network, Toronto, ON, Canada; 2 Temerty Faculty of Medicine, University of Toronto, Toronto, ON, Canada; 3 Institute of Health Policy, Management and Evaluation, University of Toronto, Toronto, ON, Canada; 4 The Wilson Centre, University Health Network and University of Toronto, Toronto, ON, Canada

**Keywords:** clinicians, communication strategies, occupational health, patient aggression, verbal de-escalation, violence prevention, workplace violence prevention (WPV)

## Abstract

**Background:**

Workplace violence (WPV) remains a significant and escalating challenge across healthcare settings, threatening the safety, well-being, and retention of frontline healthcare professionals (HCPs). Verbal de-escalation is widely promoted as a first-line strategy to prevent or manage aggressive patient behavior. However, the scope, implementation, and effectiveness of these techniques have not been consistently synthesized.

**Methods:**

This systematic review was conducted in accordance with PRISMA guidelines. Studies were eligible if they examined verbal de-escalation as a primary strategy for preventing or managing WPV in healthcare settings, including hospitals, emergency departments, psychiatric units, long-term care, and outpatient clinics. All study designs were considered, including empirical research and structured descriptive guidance relevant to clinical practice. Searches were conducted in Ovid MEDLINE ALL, Embase, Emcare, PsycINFO, and CINAHL from database inception, with results downloaded on August 29, 2024, and updated on January 30, 2026. Of 15,987 records identified, 9,096 were screened following duplicate removal, 446 underwent full-text review, and 36 studies met inclusion criteria. Methodological quality was appraised using the Mixed Methods Appraisal Tool (MMAT).

**Results:**

Substantial heterogeneity was observed in how verbal de-escalation was defined, delivered, and evaluated. Intervention studies commonly assessed structured training programs delivered through didactic sessions, interactive workshops, simulation-based education, technology-enhanced learning, or clinical unit–based training. These studies frequently reported improvements in staff knowledge, confidence, and preparedness to manage aggression. Observational and descriptive studies emphasized communication behaviors used during escalated encounters and highlighted recurring strategies, such as empathetic listening, calm verbal tone, validation, and collaborative problem-solving. Outcome measures varied considerably across studies, limiting direct comparison.

**Conclusions:**

Verbal de-escalation represents a central component of WPV prevention in healthcare, yet implementation approaches and evaluation methods remain inconsistent. The evidence suggests associations with improved clinician confidence, reduced patient agitation, and enhanced perceptions of safety. Healthcare organizations can use these findings to strengthen training curricula, integrate de-escalation within broader safety frameworks, and support sustainable workforce protection strategies.

**Systematic review registration:**

OSF, identifier https://doi.org/10.17605/OSF.IO/S7QD9.

## Introduction

### Rationale

Workplace violence (WPV) is an increasingly complex challenge in healthcare, affecting healthcare professionals (HCPs) and patient-facing staff across hospital settings. Following the onset of the COVID-19 pandemic, global WPV rates more than doubled (1–4). Recent evidence further demonstrates the magnitude of this issue, with an umbrella review reporting overall WPV prevalence among HCPs as high as 78.9% (5), while a systematic review and meta-analysis estimated a pooled prevalence of 45.0% (95% CI: 32%–58%) among female HCPs (6). These incidents are particularly pronounced in high-acuity and high-demand healthcare environments, including emergency departments, psychiatric units, and other settings where patient acuity, fast-paced workflows, and limited resources contribute to heightened risk (7–9). System-level factors, including long wait times, pervasive shortage of staff, overcrowding, underreporting of violent incidents, and lack of routine de-escalation training, can further perpetuate unsafe working conditions (10–13). Societal stressors, negative public perceptions of and reduced trust in hospitals, and receiving “bad news” related to diagnosis or prognosis can further exacerbate these risks. Limited resources for vulnerable patient populations, such as those experiencing substance use disorders, complex mental health conditions, or dual diagnoses, can further compound these risks, especially in urban hospitals where patient volumes and socioeconomic disparities are highest (13). Together, these factors, which span structural, organizational, and patient-related domains, have contributed to rising WPV rates and disproportionate physical, psychological, and occupational impacts on HCPs (14). WPV is a multifactorial phenomenon and should not be attributed solely to mental health conditions or any single patient characteristic (15). Patient aggression often occurs within broader clinical and social contexts, reinforcing the importance of patient-centered, trauma-informed approaches to workplace violence prevention and verbal de-escalation. Beyond the immediate harm to HCPs, WPV has been associated with physical injury, psychological distress, burnout, reduced job satisfaction, staff turnover, and impaired patient care, highlighting its consequences for both workforce well-being and healthcare quality (15).

In response, healthcare organizations have begun prioritizing comprehensive WPV prevention and response strategies to protect staff safety and well-being (9, 16, 17). Quality improvement (QI) initiatives increasingly focus on identifying system gaps and approaches, standardizing WPV reporting, and implementing upstream prevention measures (18–21). Within these frameworks, structured verbal de-escalation approaches have emerged as a key strategy for preventing, mitigating, and managing patient aggression before it escalates into workplace violence or physical harm, and are considered a first-line approach to WPV prevention and response (21). Verbal de-escalation strategies emphasize empathetic communication, conflict management, and partnering with patients to collaboratively reduce agitation (21). Effective de-escalation incorporates calm, professional communication, non-coercive approaches, acknowledgement of patient needs, and shared action planning alongside the patient (20, 22). Trauma-informed care models also highlight verbal de-escalation as a core strategy for supporting patients whose distress, previous trauma, or acute emotional responses may contribute to escalating behaviors (21, 23–25). By reducing escalation and preventing progression to physical violence, verbal de-escalation plays a central role in minimizing the risk of staff assault and maintaining safer care environments. Reported benefits include improved sense of patient safety, strengthened therapeutic relationships, and enhanced staff perceptions of safety and job satisfaction (9, 23, 26, 27).

Despite these benefits, multiple barriers hinder the implementation of verbal de-escalation in clinical practice. Studies identify institutional policies, limited education and training supports, and patient factors as influential barriers and facilitators to uptake, with HCPs’ reported confidence and attitudes often reflecting broader gaps in organizational preparation, rather than individual shortcomings (28, 29). These organizational gaps may also include limited visible support from senior leadership, which can result in training being perceived as a compliance-driven or ‘check-the-box’ requirement rather than as a sustained investment in frontline staff safety and skill development. Research shows that clinicians tend to match intervention type to aggression type, preferring verbal strategies for verbal aggression and restrictive measures for physical aggression, as well as emphasizing the need for training programs to promote a “talk-first” mindset (30). Additional barriers include limited availability of de-escalation training, inconsistent teaching and application of skills, inadequate resources that make restrictive practices appear more time-efficient in understaffed units, and a lack of methodologically rigorous evaluations of training interventions (30, 31). Emerging evidence also highlights the risk of overutilization of chemical and physical restraints in settings where verbal de-escalation skills are underdeveloped or inconsistently reinforced, even when de-escalation may be the most appropriate first-line response (30, 32). Studies in inpatient and acute care contexts demonstrate that enhanced de-escalation training is associated with reductions in restraint use, suggesting that reliance on restrictive interventions may reflect gaps in training and organizational support, rather than clinical necessity (32). Insufficient training leaves HCPs, particularly junior staff, vulnerable to unsafe situations (16, 27, 33). These persistent gaps in education, training, and supportive resources signal the need for a systematic examination of what verbal de-escalation techniques currently exist, and how they are defined, taught, and implemented in healthcare settings.

### Objectives

Verbal de-escalation is recognized as a central component of WPV prevention and a critical strategy for de-escalating patient behaviors. However, few reviews comprehensively examine its use, components, and effectiveness in a variety of healthcare settings. Existing literature, including (28, 31) and (29), highlights the need for high-quality evidence on verbal de-escalation training programs and clearer guidance on when and how to apply these techniques in clinical practice. Given that WPV prevention requires coordinated approaches across teams, organizational administrators, workflows, and policies, synthesizing evidence on verbal de-escalation techniques is essential to support safer, evidence-informed practice.

This systematic review aims to (i) summarize existing verbal de-escalation techniques used in healthcare settings; (ii) describe reported outcomes of these techniques in clinical practice and educational interventions; and (iii) provide evidence to inform WPV prevention and response protocols within healthcare quality improvement initiatives in Canada and internationally. Strengthening understanding of available verbal de-escalation strategies supports the broader effort to enhance WPV prevention and promote safer environments for both patients and healthcare staff.

## Methods

### Protocol and registration

This systematic review was reported in accordance with the Preferred Reporting Items for Systematic Review and Meta-Analyses (PRISMA) guidelines (34). The review was approved as part of a quality improvement initiative by the University Health Network (UHN) Quality Improvement Review Board (QI ID: 22-0499). The study protocol was registered with the Open Science Framework (OSF) [Registration DOI: 10.17605/OSF.IO/S7QD9]].

### Eligibility criteria

Studies were eligible if they focused on verbal de-escalation as a primary workplace violence prevention or management strategy in healthcare settings (e.g., hospitals, emergency departments, psychiatric units, long-term care, outpatient clinics). A broad range of evidence sources was considered, including randomized and non-randomized trials, cohort studies, cross-sectional studies, qualitative and mixed-methods studies, case studies, quality improvement initiatives, clinical guidelines, systematic or scoping reviews, and other structured guidance documents relevant to verbal de-escalation in healthcare settings. Studies were required to either report empirical outcomes related to workplace violence or provide structured descriptive guidance on verbal de-escalation approaches within healthcare settings.

Exclusion criteria included studies that:

1. Did not examine verbal de-escalation as the central intervention (e.g., focused exclusively on physical restraint training or general communication training not linked to aggression management);
2. Were conducted outside healthcare settings (e.g., law enforcement, education);
3. Were commentaries, editorials, opinion pieces, or grey literature (dissertations, conference abstracts, posters, unpublished reports);
4. Targeted patients rather than healthcare staff; or
5. Were not published in English.

### Search methods

Search strategies were developed to capture studies evaluating verbal de-escalation techniques used by HCPs to manage or prevent workplace violence. Searches were conducted in Ovid MEDLINE ALL, Embase, Emcare, PsycINFO, and EBSCO CINAHL, from database inception. Results were downloaded August 29, 2024. As the review process extended beyond the initial search date, an updated search was conducted to capture newly published evidence and ensure the review remained current, with results downloaded on January 30, 2026.

### Search

Strategies were developed for Ovid MEDLINE ALL, Embase, Emcare, PsycINFO, and EBSCO CINAHL by a health sciences librarian (MA). Ovid MEDLINE ALL was selected because it provides comparable MEDLINE coverage to PubMed while offering advanced functionality to support complex and reproducible systematic review searching. Appropriate subject headings and keywords for de-escalation of violence and verbal were used for each database. No limits for date or study type were applied during the database searches. Although no language restrictions were applied at the search stage, only studies published in English were included during screening. The full electronic search strategies for all databases are provided in **Supplementary File 1**. Search strategies were iteratively refined in consultation with the research team to ensure comprehensive retrieval of all relevant literature, across various research methodologies.

### Selection of sources of evidence

Title and abstract screening were conducted independently by two reviewers (JN, BS) using Covidence (35), which also facilitated duplicate removal. Articles that met initial inclusion criteria were advanced to full-text review. All full-text articles were independently reviewed by the same two reviewers to assess eligibility against predefined inclusion and exclusion criteria outlined in the Eligibility Criteria section. Disagreements at either stage were resolved through discussion or by consulting a third reviewer (BL). Full-text decisions were further verified by senior reviewers (BL, HS) to ensure consistency. All studies meeting inclusion criteria were advanced to data extraction and synthesis.

### Data charting process

Data from included studies were extracted using a standardized template in Covidence. Information was charted independently by two reviewers (JN, HS), with discrepancies resolved through discussion or senior reviewer verification (BL). Extracted data was updated to maintain consistency across studies and ensure accuracy.

### Data items

The data extracted included:

- Study design and setting
- Participant population (healthcare staff type, sample size, clinical context)
- Description of verbal de-escalation techniques implemented
- Outcomes measured, including staff-reported and patient-relevant metrics related to workplace violence prevention or management

### Data extraction and management

Data extraction was conducted using a standardized OneNote spreadsheet to collect information on study design, population characteristics, intervention components, implementation context, and patient-, clinician-, and organizational-level outcomes. When study details were unclear or incompletely reported, data were extracted as presented, and no additional assumptions were made. Extraction was performed by three reviewers (JN, HS, and BL), with discrepancies resolved through discussion and adjudication by a fourth reviewer (CSQ).

After satisfying the inclusion criteria, included studies were organized according to a framework of ten verbal de-escalation technique categories that were empirically derived from recurring strategies identified across the included studies following data extraction. These categories were then used to facilitate comparison across interventions. Given the substantial variation in study designs, settings, intervention formats, and outcome measures, heterogeneity was assessed descriptively through narrative synthesis rather than quantitative pooling. This categorization reflected the most salient strategies described in the literature, including strategies such as limit-setting, empathetic communication, attentive listening, and negotiation approaches. Organizing the studies in this way facilitated clearer comparison of intervention approaches and outcome patterns across healthcare settings.

In addition to content extraction, we conducted a citation network analysis of the included studies to examine inter-study connectivity within the evidence base. Bibliographic metadata were retrieved using the CrossRef API, and citation relationships were modeled as a directed network, with each edge representing one included paper citing another. Network metrics were computed in NetworkX.

### Risk of bias assessment

The methodological quality of included studies was assessed using the Mixed Methods Appraisal Tool (MMAT, 2018 version) (36). The MMAT was selected because it is specifically designed to appraise diverse study designs within a single review, including qualitative, quantitative, and mixed-methods research methodologies. It applies five core methodological criteria across five categories of study designs, allowing for consistent, comparable assessment of studies using different approaches. Two independent reviewers (HS and JN) completed all MMAT assessments, with discrepancies resolved through discussion with a third reviewer (BL). Risk-of-bias findings informed the interpretation of results and were integrated into the narrative synthesis to contextualize the strength and reliability of the evidence. As argued by Hong et al. (2018) (36), a numerical score was not given to each study, but instead papers were ranked as high, moderate, or low quality, based on MMAT criterion. All ranked studies were included in this review following quality appraisal, regardless of outcome. **Supplementary Table 1** provides an overview of the methodological quality assessment and other characteristics of the included studies.

## Results

### Selection of sources of evidence

A total of 15,987 records were identified across all databases. After removal of duplicates, 9,096 records were screened at the title and abstract stage. Of these, 446 articles were retrieved for full-text review. Following full-text assessment, 36 studies (20, 21, 25, 30, 37–68) met eligibility criteria and were included in the final synthesis (spanning qualitative, quantitative, and mixed methods designs). The study selection process is illustrated in the PRISMA flow diagram (**Figure 1**).

**Figure 1:**
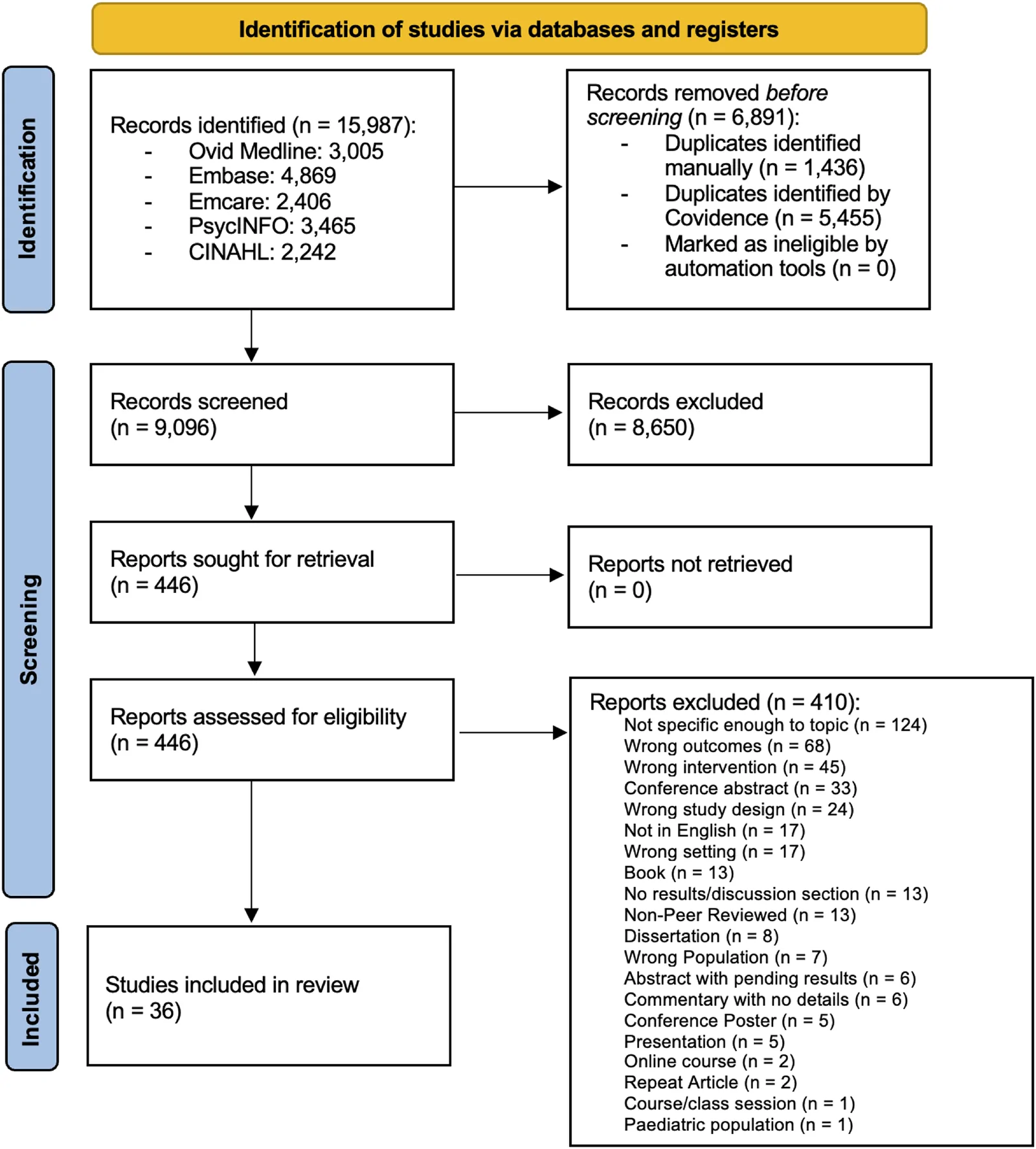
PRISMA flow diagram summarizing the study selection process.

### Characteristics of sources of evidence

The 36 included studies varied in country of origin, healthcare participant populations, study design, and the types of verbal de-escalation techniques examined. Collectively, they included nurses, physicians, medical students, and multidisciplinary clinical teams and employed qualitative, quantitative, mixed-methods, and quality improvement methodologies. **Supplementary Table 1** provides an overview of these characteristics, including the geographic distribution of studies, the clinical roles of participating HCPs, methodological approaches, and the specific verbal de-escalation strategies described within each study. Overall, the methodological quality of the included studies was generally moderate to high, with three studies assessed as low quality due to limited methodological transparency and unclear reporting across key appraisal domains (**Supplementary Table 1**).

To further examine the geographic distribution and temporal trends of the evidence, we analyzed annual publication output by country. **Figure 2** presents a heat map of annual publication counts of the included studies, visualizing variation in research activity across countries over time. Overall, publication volume increased modestly across the study period, with a small number of countries contributing the majority of publications. **Figure 3** displays the cumulative global distribution of publications, highlighting concentration of research output in specific regions.

**Figure 2:**
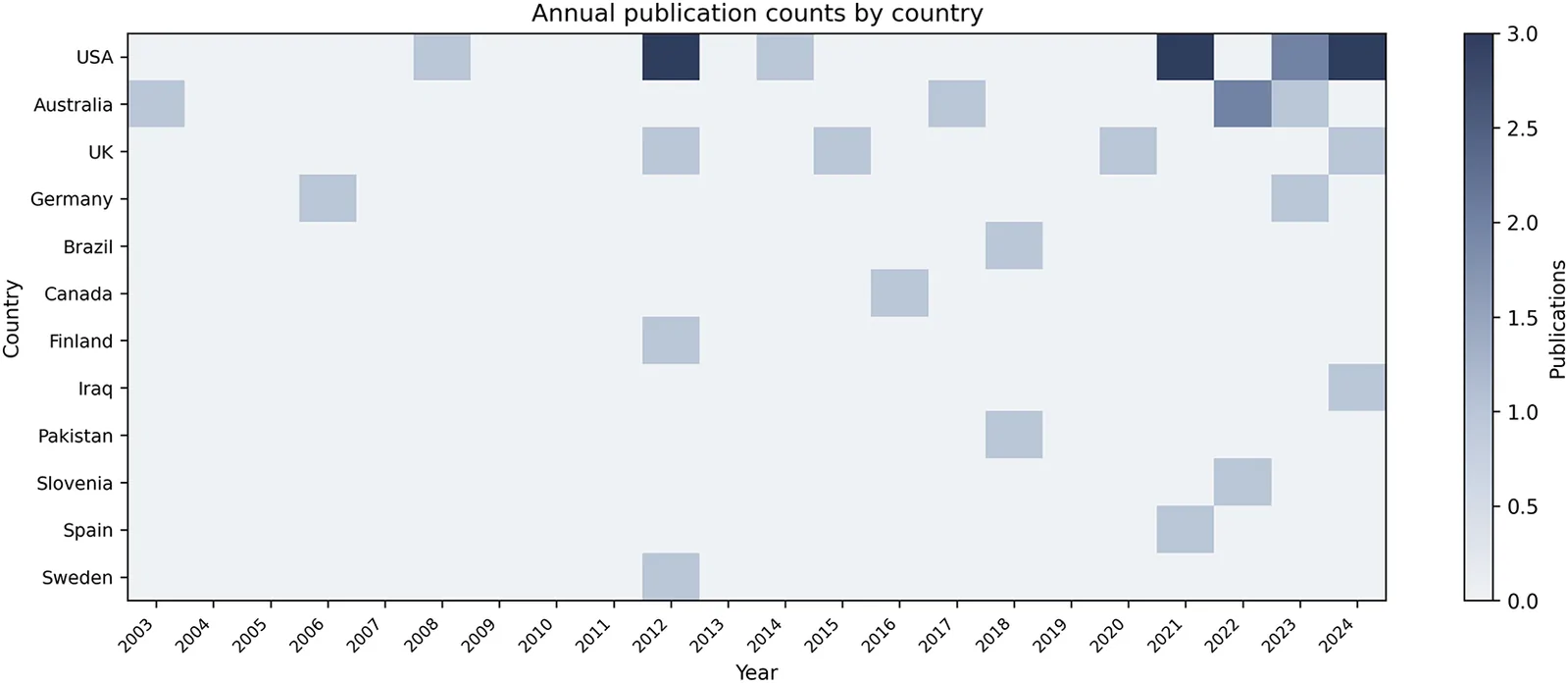
Heat map of annual publication counts by country. Rows represent countries and columns represent publication years; darker cell shading indicates a greater number of included studies published in a given country and year.

**Figure 3:**
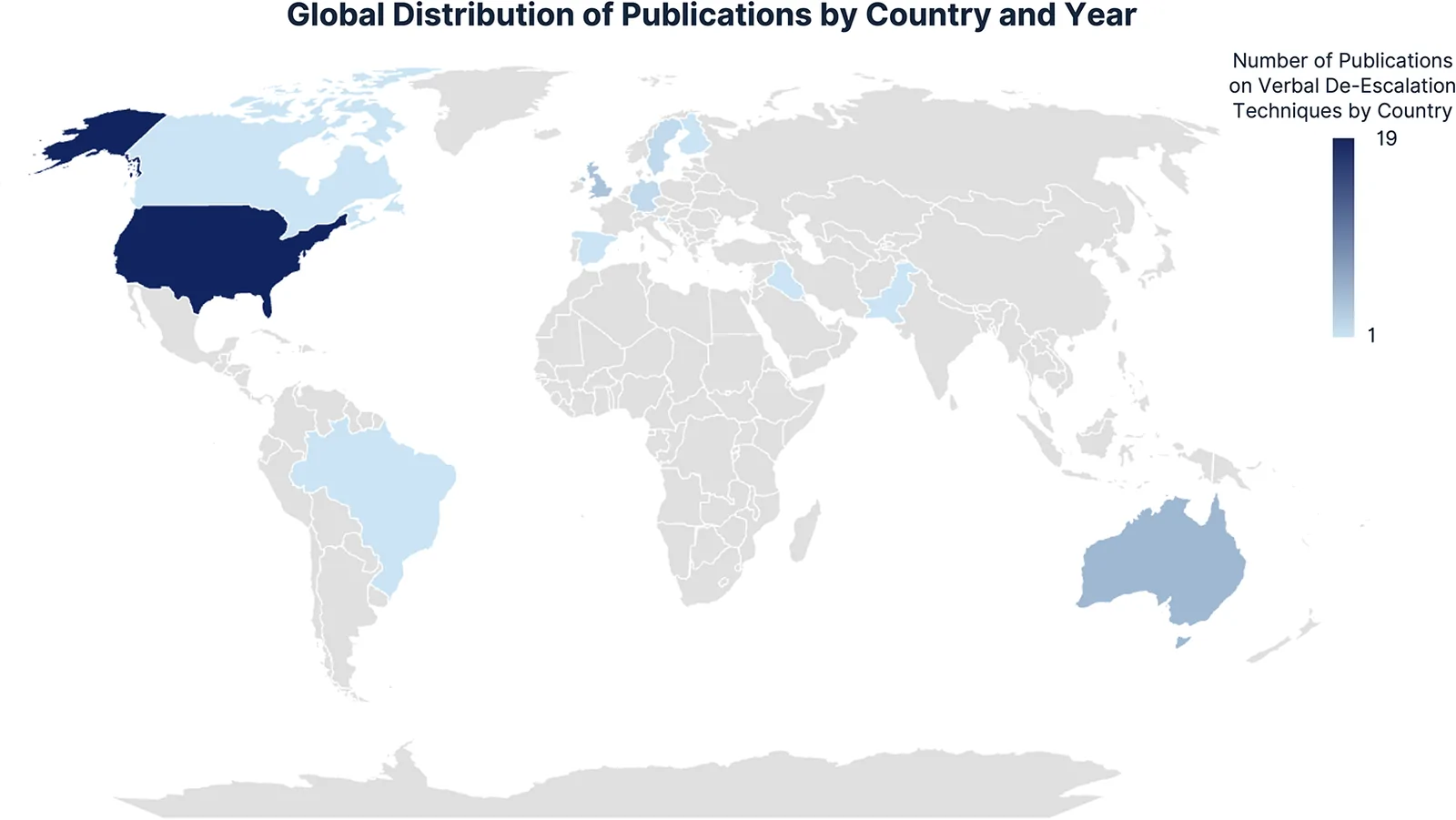
World map showing total publication counts by country. Countries are shaded according to the cumulative number of included studies across the study period; darker shading indicates a greater number of publications, while gray indicates no included publications.

To characterize the bibliometric structure of the field, we conducted a citation network analysis of the included studies (**Figure 4**). Of the 36 included studies, 34 had resolvable DOIs; using CrossRef data we constructed a directed network in which each edge represents one included paper citing another. The network comprised 34 nodes and 31 internal citations, a density of 2.76%, and resolved into 14 components: a 19-paper main component, a separate 3-paper component, and 12 isolated papers. Only 10 papers (29.4%) were cited by another study in the set, and 12 (35.3%) were connected in neither direction. Citation attention was dominated by a single reference: Richmond et al. (21) accounted for 12 of the 31 internal citations (38.7%), followed by Cowin et al. (41) with 5, and by Hallett et al. (30), Mavandadi et al. (47) and Price et al. (50) with 3 each.

**Figure 4:**
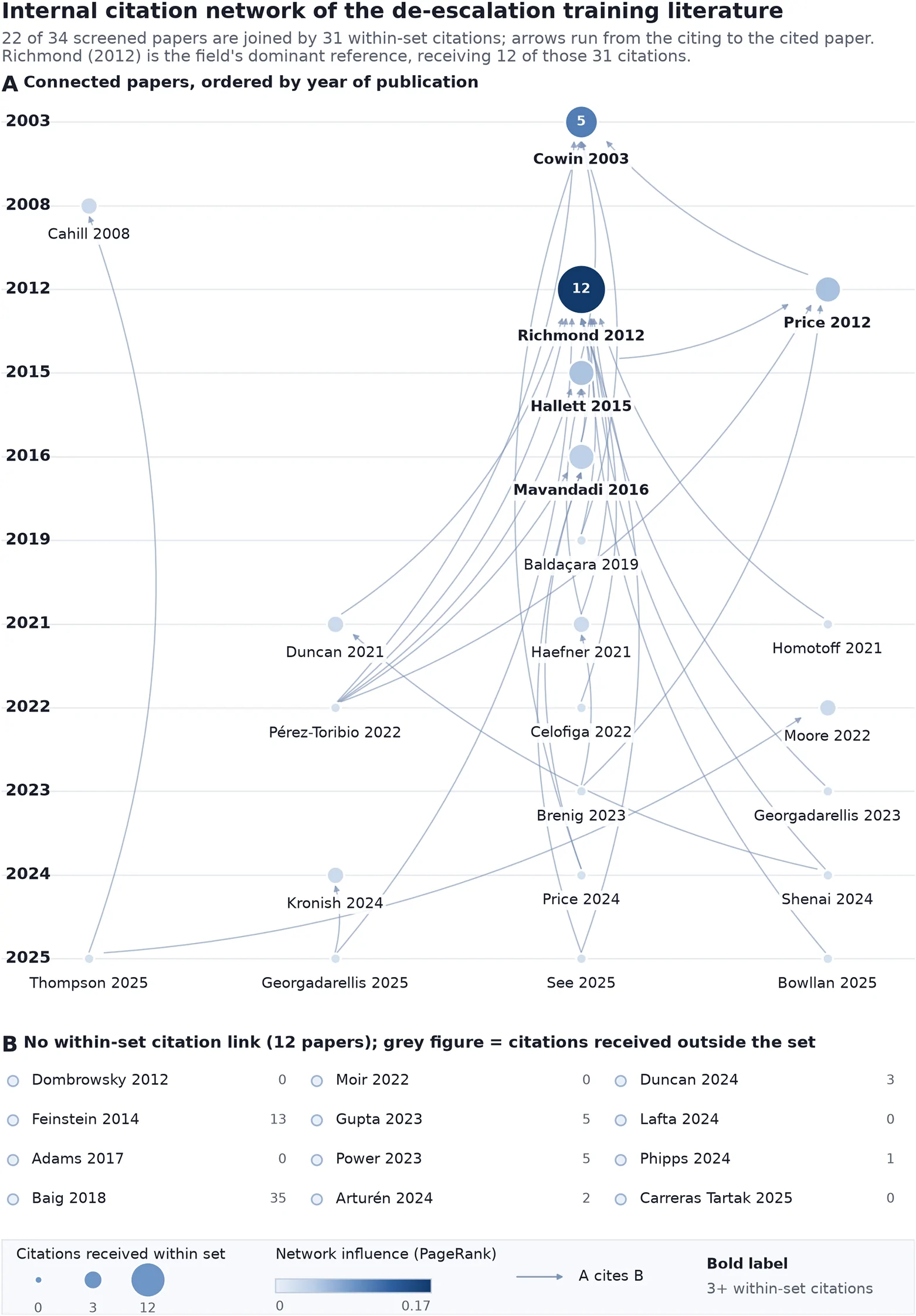
Internal citation structure of the included de-escalation and workplace violence literature (n = 34). **(A)** The 22 papers connected by at least one within-set citation, arranged in rows by year of publication; rows are ordinally spaced rather than drawn to a linear time scale. Arrows run from the citing paper to the paper it cites (n = 31 internal citations). Node area is proportional to the number of citations a paper received from other papers in the set, and fill darkness encodes PageRank. Horizontal position is assigned to minimize arc length and label overlap and carries no substantive meaning. Two multi-paper components are present: a 19-paper main component and a separate 3-paper component (Thompson 2025 citing Cahill 2008 and Moore 2022, at left). **(B)** The 12 papers with no within-set citation link, each annotated with the number of citations received from outside the set. Citation relationships were retrieved from the CrossRef API; network metrics were computed in NetworkX.

To further contextualize the evidence base, **Table 1** summarized the 10 verbal de-escalation technique categories empirically derived from the included literature, providing a concise description of each and its associated patient, clinician, and organizational outcomes. The table was structured to facilitate straightforward interpretation, enabling readers to identify the outcomes reported in the included studies.

**Table 1:** Summary of verbal de-escalation techniques and associated patient, clinician, and organizational outcomes.

Verbal de-escalation technique	Description	Associated outcomes (patient/clinician/organization)	Underlying theoretical model	Disciplinary field	Literature source
#1 Limit-setting and establishing boundaries	Firmly set realistic limits for acceptable behaviors; knowing when to exert control, promote trust and accountability between patient and clinician	- Patient: Reduced agitation, clearer behavioral expectations - Clinician: Increased confidence in managing aggression - Organization: Fewer escalated incidents, improved safety	Behavioral and social learning models of contingency management and boundary-setting; therapeutic limit-setting within milieu therapy	Clinical psychology; psychiatric nursing; mental health services; psychiatry	(21, 38, 39, 50, 52, 57, 60, 66)
#2 Empathetic tone and pace of speech	Speak in a calm, firm, and measured tone even when emotions are heightened	- Patient: Feeling heard, reduced emotional escalation - Clinician: Maintains control and reduces stress - Organization: Supports therapeutic environment	Client-centered (humanistic) communication theory and affect regulation; crisis intervention and de-escalation frameworks	Clinical psychology; counselling; communication studies; psychiatric nursing	(21, 25, 37–42, 48, 50–54, 56, 60, 67)
#3 Attentive listening approach	Listen fully to patient concerns without interrupting; focus on understanding content and underlying meaning; avoid projecting emotions	- Patient: Increased satisfaction, reduced frustration - Clinician: Better understanding of triggers, improved engagement - Organization: Fewer misunderstandings leading to incidents	Active listening and empathic communication models; person-centered counselling and therapeutic alliance frameworks	Clinical psychology; counselling; communication studies; psychiatric nursing	(21, 25, 37–45, 47, 49–54, 56, 60, 68)
#4 Verbalizing intentions to collaboratively de-escalate	Clearly communicate intent to maintain safety and work with the patient; introduce yourself and supporting staff	- Patient: Increased cooperation, perceived safety - Clinician: Clearer communication, reduced tension - Organization: Smoother resolution of incidents	Collaborative problem-solving and shared decision-making models; relational and therapeutic alliance frameworks	Clinical psychology; psychiatry; psychiatric nursing; communication studies	(20, 25, 38–40, 45, 46, 55, 56, 58, 59, 63)
#5 Repeating instructions and concerns	Reinforce limits, choices, and care alternatives; account for comprehension difficulties during agitation	- Patient: Better understanding, decreased confusion - Clinician: More effective communication, fewer repeated conflicts - Organization: Reduced errors or miscommunication	Cognitive–behavioral and information-processing models; health communication theory related to message clarity and redundancy	Clinical psychology; health communication; psychiatric nursing	(21, 43, 44, 52, 57, 61, 66)
#6 Developing open communication channels	Encourage patient to share emotions; provide feedback; use open-ended questions; measured self-disclosure by staff	- Patient: Greater trust and self-expression - Clinician: Enhanced insight into patient needs - Organization: Supports culture of patient-centered care	Patient-centered communication, therapeutic alliance, and motivational interviewing–influenced engagement models	Clinical psychology; counselling; communication studies; psychiatric nursing	(20, 21, 30, 42, 49–54, 61, 64)
#7 Appropriate negotiation tactics	Remain flexible and reasonable; honor patient requests when possible; involve third parties if needed	- Patient: Higher satisfaction, sense of fairness - Clinician: Reduced frustration, improved conflict resolution skills - Organization: Decreased escalation and complaints	Conflict resolution and negotiation theory; procedural justice and collaborative problem-solving models	Social psychology; criminology/conflict management; communication studies; psychiatric nursing	(39–41, 43, 44, 54, 55, 59)
#8 Offering treatment choices or amenities	Provide small accommodations (snacks, drinks, activities, comfort items) to support de-escalation	- Patient: Reduced agitation, improved engagement - Clinician: Facilitates cooperation without coercion - Organization: Supports patient-centered care practices	Behavioral reinforcement and autonomy-supportive care models; self-determination theory (choice and control)	Clinical psychology; behavioral medicine; nursing; health services research	(21, 41, 45)
#9 Offering cool down periods or relocating the patient	Provide brief time-outs or move patient away from stressors to calm emotions	- Patient: Emotional regulation, reduced aggressive behavior - Clinician: Increased safety, opportunity to reassess - Organization: Prevents incidents from escalating, safer environment	Arousal reduction and emotion-regulation models; behavioral time-out and environmental stress theories	Clinical psychology; psychiatry; psychiatric nursing; environmental/health psychology	(43, 44, 56, 57)
#10 Debriefing after the escalated incident	Discuss the incident with patient and family; establish non-violent methods for expressing emotions	- Patient: Understanding and reflection, improved therapeutic relationship - Clinician: Insight into triggers, learning opportunity - Organization: Continuous improvement in workplace violence prevention	Critical incident stress debriefing and reflective practice models; learning-from-events and quality improvement theory	Clinical psychology; psychiatry; psychiatric nursing; health services/quality improvement	(21, 58, 59, 63, 67)

### Results of individual sources of evidence

The included evidence consisted of both studies that described existing verbal de-escalation practices within clinical care and studies that evaluated verbal de-escalation as a structured intervention. Descriptive studies highlighted common communication strategies used during escalation, while intervention studies assessed training effectiveness using validated tools or incident-related outcomes. Verbal de-escalation techniques across studies aligned with ten core categories summarized in **Table 1**. Since studies varied widely in how they described or measured verbal de-escalation techniques, and no standard reporting metric existed across all included studies, **Table 1** groups the techniques into qualitative categories (patient, clinician, organization) that reflect the recurring communication strategies found across sources.

### Synthesis of results

The synthesis revealed substantial heterogeneity in how verbal de-escalation was defined, delivered, and evaluated across the included studies. Despite differences in study design, intervention format, educational approach, and outcome measures, consistent patterns emerged in both the communication strategies described and the outcomes assessed. Intervention studies primarily evaluated structured verbal de-escalation training programs and frequently reported improvements in staff knowledge, confidence, and preparedness. In contrast, descriptive and observational studies focused on communication behaviors used during escalated patient encounters and the practical application of verbal de-escalation techniques in clinical settings.

Beyond heterogeneity in study design and outcomes, a great deal of variation was observed in the educational methods used to teach verbal de-escalation. Across intervention studies, training formats ranged from didactic instruction and interactive workshops to simulation-based education, technology-enhanced learning, and clinical unit–based learning. To clarify the educational approaches underpinning this literature and strengthen interpretation of training-related outcomes, we categorized included studies by their primary educational method or format (**Table 2**).

**Table 2:** Overview of educational approaches used in verbal de-escalation training.

Educational method	Key features	Studies using this method	Brief evidence summary
Didactic or curriculum-based instruction	Classroom-based and structured teaching sessions (may take the form of lectures, guided discussion, or conceptual frameworks)	(54, 55, 60, 67, 68)	Used mainly to introduce core concepts, communication principles, and frameworks for de-escalation. Studies consistently reported improvements in HCP knowledge, confidence, and perceived preparedness, especially when delivered early in formal training.
Interactive or experiential learning	Role-play, facilitated discussion, group reflection, and applied exercises	(51, 55, 60, 62, 63, 68)	Interactive activities allowed learners to practice and reflect on de-escalation skills and were usually delivered as part of a broader training program, rather than delivered as stand-alone interventions.
Simulation-based education (standardized patient or scenario-based)	Semi-structured simulated encounters with debriefing	(54, 60–64, 67, 68)	Simulation allowed learners to practice verbal de-escalation in realistic, emotionally-charged scenarios. Across studies, simulation was associated with increased confidence and self-efficacy, although objective performance differences were seen less consistently.
Technology-enhanced simulation (virtual reality)	Immersive VR environments with conversational agents and automated feedback	(48)	VR-based training was feasible and acceptable to clinicians, allowing for repeated practice and objective interaction tracking. Findings emphasized usability, realism, and accessibility, rather than clinical effectiveness outcomes.
Clinically-embedded education	Education delivered in real clinical settings, often by experts, using live cases or unit-specific scenarios	(51, 58, 66)	*In-vivo* training supported application to practice and was associated with greater use of verbal de-escalation and fewer violent incidents, with smaller gains in staff confidence.
Assessment-focused simulation (measurement)	Simulated encounters used to evaluate de-escalation skills rather than teach them	(47)	Simulation was used to validate a quantitative de-escalation measurement tool, contributing to the evidence base on how de-escalation behaviors can be operationalized and assessed.
Observational/descriptive (non-educational)	Surveys, chart reviews, or vignettes examining de-escalation practices without an intervention	(30, 49)	These studies did not evaluate training but highlighted variability in how de-escalation is understood and applied in practice, highlighting the need for clearer educational guidance and standardized training approaches.

Across sources, verbal de-escalation techniques clustered into ten consistent categories (**Table 1**), reflecting shared elements of empathic communication, common behavioral responses, environmental awareness, limit-setting, and collaborative problem-solving. Outcomes similarly aligned into three broad domains: (i) clinician-focused (confidence, perceived competence, stress responses), (ii) patient-focused (incident severity, recurrence, agitation levels), and (iii) organizational-level outcomes (restraint use, Code White activations [i.e., institutional emergency responses to violent or aggressive incidents], safety). These three domains were identified by categorizing reported outcomes from the included studies according to the level at which the effects of verbal de-escalation were measured (clinician, patient, or organizational). This approach aligns with the inclusion criteria for this systematic review, which required studies to report empirical workplace violence–related outcomes or provide structured descriptive guidance on verbal de-escalation approaches. Organizing outcomes into clinician-, patient-, and organizational-level domains enabled synthesis across heterogeneous study designs within a common outcome framework. Methodological diversity, ranging from simulation and standardized patient assessment to real-world incident monitoring and educational programs, allowed verbal de-escalation techniques to be examined both as skills acquired through training and as strategies rehearsed during routine patient-facing care.

## Discussion

### Summary of evidence

Across the 36 studies included in this review, verbal de-escalation was consistently described and evaluated as a beneficial approach to managing WPV in healthcare settings, with no evidence suggesting it caused harm when applied appropriately. However, the effectiveness of these techniques was sometimes context-dependent and subject to caveats: certain trainee groups, such as final-year medical students in specific tracks (e.g. internal medicine), reported lower confidence and preparedness in using verbal de-escalation (54); time constraints in busy clinical settings may limit the opportunity to de-escalate effectively, although even brief interventions are generally considered worthwhile (21); aggressive limit-setting may exacerbate behavior if used in isolation rather than alongside autonomy-supportive strategies (50); some patients, specifically those requiring mechanical restraint, may not respond to prior verbal de-escalation (49); and in specific settings such as forensic psychiatry, evidence on the impact of staff training on aggression reduction remains limited and definitions of de-escalation vary (39).

Despite these contextual considerations, the included studies consistently highlighted a set of core verbal de-escalation techniques that formed the basis of training and intervention programs. Across studies, despite variations in terminology, these techniques clustered around therapeutic communication, active and reflective listening, empathetic engagement, maintaining a calm tone and demeanor, and clear limit-setting. These techniques align with the ten categories synthesized in **Table 2**, with the most frequently reported being: Empathetic tone and pace of speech, attentive listening approach, and verbalizing intentions to collaboratively de-escalate. These skills were described across both stand-alone HCP training programs (37, 44, 49) and multicomponent interventions that paired verbal de-escalation with organizational supports such as reporting systems or environmental modifications (25, 39, 55).

Notably, studies that reported more substantial reductions in WPV typically implemented verbal de-escalation within multicomponent interventions. For example, this looked like pairing de-escalation training with structural supports like violence reporting mechanisms, team-based response protocols, or clear organizational policies (25, 51, 55). In these cases, improvements likely reflected not only enhanced individual skill, but also system-level reinforcements that enabled consistent use of verbal de-escalation techniques. This finding echoes WPV reduction systematic review recommendations emphasizing that WPV prevention require integrated approaches combining structured policies and environmental changes as they are more likely to help decrease WPV rates (69). These findings also have implications for implementation. Integrating verbal de-escalation into routine practice may require ongoing organizational support, clear protocols, and reinforcement of skills beyond initial training. These supports may help promote consistent use of verbal de-escalation in practice.

Collectively, these findings suggest that structured verbal de-escalation training may strengthen HCPs’ confidence, perceived competence, and communication self-efficacy, potentially improving their ability to recognize and respond to escalating situations before they progress to violence (21, 38, 43). The reported patient-level benefits, including reduced agitation, shorter escalation duration, and improved patient–clinician trust, further suggest that effective verbal de-escalation may contribute to safer and more therapeutic clinical encounters (40, 46, 56). Although organizational-level outcomes were examined less frequently, the available evidence indicates that verbal de-escalation is likely to be most effective when implemented within broader workplace violence prevention frameworks that include complementary organizational supports, rather than as a stand-alone intervention (20, 51). Taken together, these findings reinforce verbal de-escalation as a foundational skill for frontline HCPs; however, maximizing its impact on workplace violence prevention, staff well-being, and workforce retention requires sustained organizational investment and system-level implementation (53). The citation network analysis further suggests that the de-escalation literature has developed largely in parallel rather than cumulatively. The sparse, hub-dependent structure, with citation attention concentrated around a small number of influential papers and consensus statements, indicates limited connectivity across the empirical evidence base.

### Broader implications

Findings from this review suggest that verbal de-escalation should not be viewed solely as an individual communication technique, but as a practice that may be influenced by broader clinical, organizational, and social contexts. Episodes of patient aggression often occur within complex clinical and social circumstances, including unmet emotional needs, acute medical or psychiatric conditions, substance use, or broader social stressors such as poverty, racism, and chronic exposure to trauma (4, 70, 71). These contextual factors reinforce the importance of approaches that emphasize empathy, trauma-informed care, contextual understanding, and collaborative communication when appropriate. Consistent with this, the included studies suggest that techniques centered on validating emotions, reducing perceived threat, and prioritizing patient autonomy may be more effective than approaches relying primarily on behavioral control or risk-based assumptions (47, 52).

Embedding verbal de-escalation within broader organizational workplace violence prevention strategies has important implications for HCPs and organizations. HCPs from equity-deprived groups face disproportionate discrimination, emotional labor, and psychological strain, which can heighten distress during volatile encounters (72). Training that acknowledges the lived realities of HCPs, while situating verbal de-escalation within broader conflict management and organizational responsibilities, can foster psychological safety, reduce burden on individual HCPs, and support more consistent implementation of verbal and complementary non-verbal de-escalation skills. However, a recent Cochrane review highlighted the dearth of evidence regarding the efficacy of training programs in reducing WPV (73). Thus, optimizing the impact of de-escalation training also requires complementary efforts to address structural inequities, strengthen reporting processes, and cultivate leadership cultures that do not view violence as an inevitable part of the job. A social justice–oriented approach positions verbal de-escalation as both an immediate harm-reduction tool and a long-term strategy for fostering safer and more accountable healthcare environments for everyone.

Recent synthesis work further supports these findings. An integrative review examining de-escalation training among nurses, specifically in emergency care settings, found that such training consistently improved confidence, communication skills, empathy, and perceived preparedness, while demonstrating more variable effects on the frequency and severity of violent incidents (74). Importantly, this review emphasized that environmental and systemic stressors, such as overcrowding, time pressure, and limited staffing, were frequent antecedents of aggression, and that organizational support strongly moderated training effectiveness. These findings align with our review’s observation that verbal de-escalation is most effective when embedded within supportive institutional contexts.

However, verbal de-escalation to address WPV has important limitations and cannot be relied on as a single, universal approach. Practical implementation may be influenced by patient, clinician, and organizational factors. For example, language barriers, cultural differences, and communication impairments may limit the effectiveness of verbal strategies and hinder the establishment of rapport (75). In addition, in situations of imminent harm or weapon involvement, verbal techniques alone may be unsafe and rapid activation of team-based, physical, and pharmacologic responses may be required to protect staff and patients (49). In these circumstances, verbal de-escalation should be viewed as one component of a broader conflict management approach that may also incorporate non-verbal de-escalation strategies and other appropriate safety interventions. As well, when patients are severely agitated, intoxicated, or experiencing significant delirium or psychosis, they may be unable to process or respond to verbal cues, reducing the feasibility of de-escalation as a primary strategy (73). Furthermore, the studies included in this review primarily focused on in-person patient interactions within healthcare settings and did not explicitly examine other forms of workplace violence, such as out-of-hospital incidents, online harassment, or stalking of HCPs.

Emerging evidence helps clarify when verbal de-escalation is more or less likely to be effective. A recent mixed-methods analysis of hospital workplace violence reports described a subgroup of incidents characterized by denial, minimization, or justification of harmful behavior in the absence of identifiable medical impairment (76). In incidents characterized by these behaviors, verbal violence was common, and many incidents required formal safety responses, such as activation of security personnel, behavioral emergency teams, or institutional workplace violence protocols, suggesting limited responsiveness to empathic or autonomy-supportive verbal strategies alone. In contrast, verbal de-escalation appears more effective when aggression arises from distress, confusion, or impaired impulse control. Differentiating the underlying causes of aggression, such as medical illness, cognitive impairment, or intoxication, may help HCPs select more appropriate de-escalation approaches, recognize when escalation to additional safety interventions is warranted, and guide post-incident support and training following workplace violence events.

Despite our results supporting the effectiveness of verbal de-escalation training for HCPs, it is crucial to recognize that WPV is a multi-faceted issue that requires complex, system-level interventions to effectively reduce its impact (4, 69). Within WPV prevention training for HCPs, education on non-verbal de-escalation, safe use of physical and chemical restraints, trauma-informed care, and inclusive care has also been described as important and potentially beneficial components of comprehensive programs (77). However, education represents only one part of a broader workplace violence prevention strategy, which also requires organizational policies, operational supports, technology, and environmental interventions to achieve meaningful reductions in WPV (4, 69). Consequently, these findings should be interpreted as guidance for one component of a broader system-level workplace violence prevention approach, rather than as support for a single intervention to independently reduce WPV in healthcare (4, 78, 79).

Future research should examine the long-term effectiveness of verbal de-escalation techniques in healthcare settings, including extended follow-up periods to assess sustained impacts on clinician, patient, and organizational outcomes, as well as the extent to which increases in de-escalation knowledge and awareness are maintained over time. Studies could also compare verbal de-escalation with broader workplace violence prevention approaches, including combinations of verbal, non-verbal, and pharmacological strategies, across different clinical contexts, and examine whether other forms of workplace violence, such as out-of-hospital incidents, online harassment, or stalking of HCPs, require different prevention and response strategies. An emerging area for investigation is the development and evaluation of AI-powered, virtual simulation-based training tools that could provide standardized and interactive opportunities for clinicians to practice verbal de-escalation techniques and receive real-time feedback. Additionally, given that many existing studies were limited by convenience or volunteer sampling, employing random or representative sampling strategies in future studies could strengthen the external validity (generalizability) of findings. When future systematic reviews are conducted, efforts should be made to minimize the inclusion of studies with high risk of bias wherever possible since these studies can substantially affect certainty of evidence evaluations.

### Limitations

This systematic review has several limitations related to its design and methodology. First, the search was limited to four electronic databases and restricted to studies published in English, which may have excluded relevant research published in other languages or in the grey literature. Second, included studies were heterogeneous in terms of design, setting, and outcomes, which limited the comparability of interventions and precluded meta-analysis. Third, many studies focused on single institutions, educational programs, or departments, reducing the generalizability of findings to other healthcare contexts. Fourth, the majority of evidence from the included studies comes from observational or pre-post studies, which means reported associations between verbal de-escalation and improved outcomes cannot be interpreted as causal. While the findings suggest verbal de-escalation is linked to positive benefits for patients, clinicians, and organizations, the results should be interpreted as correlational, and other confounding factors may also contribute to observed outcomes in workplace violence management.

## Conclusion

WPV continues to pose a significant challenge for healthcare systems, threatening the safety and well-being of frontline HCPs. Effective prevention strategies, including verbal de-escalation techniques, are important for supporting safer interactions and improving outcomes for both patients and HCPs. This systematic review synthesized the existing literature to identify core verbal de-escalation techniques and mapped their reported outcomes across clinician, patient, and organizational domains. The evidence suggests these techniques are associated with improved clinician confidence, reduced patient agitation, and enhanced organizational safety. Healthcare organizations, hospital administrators, and safety officers can leverage these findings to develop and scale training curriculum, integrate verbal de-escalation into broader safety frameworks, and strengthen workforce preparedness.

## Data Availability

The original contributions presented in the study are included in the article/**Supplementary Material**. Further inquiries can be directed to the corresponding author.

## Supplementary material

Supplementary Table 1 Characteristics of included studies (including MMAT quality appraisal ratings).

Supplementary File 1 Full electronic database search strategies.

Supplementary File 2 Extended theoretical and disciplinary framework mapping of verbal de-escalation techniques.

Supplementary File 3 PRISMA 2020 Checklist.

## References

[1] McGuire SS Finley JL Gazley BF Mullan AF Clements CM . The team is not okay: Violence in emergency departments across disciplines in a health system. West J Emerg Med. (2023) 24:169–77. doi: 10.5811/westjem.2022.9.57497 36976596 PMC10047752

[2] Brigo F Zaboli A Rella E Sibilio S Canelles MF Magnarelli G . The impact of COVID-19 pandemic on temporal trends of workplace violence against healthcare workers in the emergency department. Health Policy. (2022) 126:1110–6. doi: 10.1016/j.healthpol.2022.09.010 36171162 PMC9502437

[3] Jones CB Sousane Z Mossburg S . Addressing workplace violence and creating a safer workplace Addressing Workplace Violence Creat Safer Workplace (2023). Available online at: https://psnet.ahrq.gov/perspective/addressing-workplace-violence-and-creating-safer-workplace (Accessed February 10, 2026).

[4] Schulz-Quach C Lyver B Reynolds C Hanagan T Haines J Shannon J . Understanding and measuring workplace violence in healthcare: A Canadian systematic framework to address a global healthcare phenomenon. BMC Emerg Med. (2025) 25:9. doi: 10.1186/s12873-024-01144-1 39800705 PMC11727261

[5] Rossi MF Beccia F Cittadini F Amantea C Aulino G Santoro PE . Workplace violence against healthcare workers: An umbrella review of systematic reviews and meta-analyses. Public Health. (2023) 221:50–9. doi: 10.1016/j.puhe.2023.05.021 37406450

[6] Ajuwa MEP Veyrier CA Cousin Cabrolier L Chassany O Marcellin F Yaya I . Workplace violence against female healthcare workers: A systematic review and meta-analysis. BMJ Open. (2024) 14:e079396. doi: 10.1136/bmjopen-2023-079396 39209501 PMC11369783

[7] Fawzy Y Siddiqui Z Narouze S Potru S Burgart AM Udoji MA . Violence in the pain clinic: The hidden pandemic. Reg Anesth Pain Med. (2023) 48:387–91. doi: 10.1136/rapm-2022-104308 36754543

[8] Avillion AE Mitus MC . Workplace violence and safety. (2023).

[9] Sanders J Thomas L Kerr T Benita T Abela K Ulrich B . Interventions to prevent and mitigate workplace violence: Teaming up for a safer environment. Nurs Manag (Harrow). (2023) 54:20–30. doi: 10.1097/nmg.0000000000000040 37527648

[10] d’Ettorre G Pellicani V Mazzotta M Vullo A . Preventing and managing workplace violence against healthcare workers in emergency departments. Acta BioMed Atenei Parm. (2018) 89:28–36. doi: 10.23750/abm.v89i4-S.7113 29644987 PMC6357631

[11] He Y Holroyd E Koziol-McLain J . Understanding workplace violence against medical staff in China: A retrospective review of publicly available reports. BMC Health Serv Res. (2023) 23:660. doi: 10.1186/s12913-023-09577-3 37340402 PMC10283279

[12] Ayasreh IR Hayajneh FA . Workplace violence against emergency nurses: A literature review. Crit Care Nurs Q. (2021) 44:187–202. doi: 10.1097/CNQ.0000000000000353 33595966

[13] Keith MM . Code White: Sounding the Alarm on Violence Against Health Care Workers. Toronto: Between the Lines (2021). p. 1–.

[14] Sahebi A Golitaleb M Moayedi S Torres M Sheikhbardsiri H . Prevalence of workplace violence against health care workers in hospital and pre-hospital settings: An umbrella review of meta-analyses. Front Public Health. (2022) 10:895818. doi: 10.3389/fpubh.2022.895818 36003634 PMC9393420

[15] Fiorino FS Oliveri C Vivarelli S Fenga C . The rising tide of workplace violence in the healthcare sector: A global scoping review of burden, determinants, and prevention strategies (2021–2025). Front Public Health. (2026) 14:1851734. doi: 10.3389/fpubh.2026.1851734 42221657 PMC13216234

[16] Stephen T King K Taylor M Jackson M Hilario C . A virtual, simulated code white for undergraduate nursing students. Can J Nurs Res. (2022) 54:320–30. doi: 10.1177/08445621221101290 35578409 PMC9379387

[17] Reißmann S Wirth T Beringer V Groneberg DA Nienhaus A Harth V . I think we still do too little”: Measures to prevent violence and aggression in German emergency departments – a qualitative study. BMC Health Serv Res. (2023) 23:97. doi: 10.1186/s12913-023-09044-z 36717889 PMC9885053

[18] Caravella RA Ying P Siegel C Vaughn R Deutch AB Caroff A . Quality improvement framework to examine health care disparities in behavioral emergency management in the inpatient medical setting: A consultation-liaison psychiatry health equity project. J Acad Consult-Liaison Psychiatry. (2023) 64:322–31. doi: 10.1016/j.jaclp.2023.04.002 37060945

[19] Lyver B Gorla J Schulz-Quach C Anderson M Singh B Hanagan T . Identifying quality indicators to measure workplace violence in healthcare settings: A rapid review. BMC Emerg Med. (2024) 24:29. doi: 10.1186/s12873-024-00943-w 38360571 PMC10870575

[20] Haefner J Dunn I McFarland M . A quality improvement project using verbal de-escalation to reduce seclusion and patient aggression in an inpatient psychiatric unit. Issues Ment Health Nurs. (2021) 42:138–44. doi: 10.1080/01612840.2020.1789784 32749904

[21] Richmond J Berlin J Fishkind A Holloman G Zeller S Wilson M . Verbal de-escalation of the agitated patient: Consensus statement of the American Association for Emergency Psychiatry Project BETA De-escalation Workgroup. West J Emerg Med. (2012) 13:17–25. doi: 10.5811/westjem.2011.9.6864 22461917 PMC3298202

[22] Robbins KC . De-escalation in health care. Nephrol Nurs J. (2019) 46:345–6.

[23] Endriyani S Pastari M Martini S . The influence of de-escalation techniques on reducing anger responses in drug clients. Formosa J Sci Technol. (2023) 2:1465–80. doi: 10.55927/fjst.v2i6.4705

[24] González-Rodríguez A Seeman MV Román E Natividad M Pagés C Ghigliazza C . Critical issues in the management of agitation, aggression, and end-of-life in delusional disorder: A mini-review. Healthcare. (2023) 11:458. doi: 10.3390/healthcare11040458 36832992 PMC9956049

[25] Gupta I Eid SM Harris CM Kauffman R Washburn C Singh A . Prevalence of workplace violence and effects of de-escalation training among hospitalists: A pilot study. J Community Hosp Intern Med Perspect. (2023) 13. doi: 10.55729/2000-9666.1189 37877044 PMC10593168

[26] Solorzano Martinez AJ De Oliveira GC . Workplace violence training programs for nursing students: A literature review. J Am Psychiatr Nurses Assoc. (2021) 27:361–72. doi: 10.1177/1078390321994665 33645347

[27] Millar J Andargache E Oliver N . A different state of mind: Developing a mental health simulation programme for foundation doctors in Scotland. Int J Healthc Simul. (2022):HOUK2878. doi: 10.54531/HOUK2878

[28] Price O Baker J Bee P Grundy A Scott A Butler D . Patient perspectives on barriers and enablers to the use and effectiveness of de‐escalation techniques for the management of violence and aggression in mental health settings. J Adv Nurs. (2018) 74:614–25. doi: 10.1111/jan.13488 29082552

[29] Krull W Gusenius TM Germain D Schnepper L . Staff perception of interprofessional simulation for verbal de-escalation and restraint application to mitigate violent patient behaviors in the emergency department. J Emerg Nurs. (2019) 45:24–30. doi: 10.1016/j.jen.2018.07.001 30122301

[30] Hallett N Dickens GL . De‐escalation: A survey of clinical staff in a secure mental health inpatient service. Int J Ment Health Nurs. (2015) 24:324–33. doi: 10.1111/inm.12136 25975221

[31] Price O Baker J Bee P Lovell K . The support-control continuum: An investigation of staff perspectives on factors influencing the success or failure of de-escalation techniques for the management of violence and aggression in mental health settings. Int J Nurs Stud. (2018) 77:197–206. doi: 10.1016/j.ijnurstu.2017.10.002 29100202

[32] Orlick A . Reducing the Use of Physical and Chemical Restraints Through Enhanced De-Escalation Training in Adult Inpatient Psychiatry (2020). United States -- Pennsylvania: Wilkes University. Available online at: https://www.proquest.com/docview/2455788750/abstract/4F4B8DD775E44F02PQ/1 (Accessed February 10, 2026). D.N.P.

[33] Wigozki E Sullivan S Jacobo M . Dedicated de-escalation training for nurse navigators. Oncol Nurs Forum. (2024) 51:207C.

[34] Moher D Liberati A Tetzlaff J Altman DG PRISMA Group . Preferred reporting items for systematic reviews and meta-analyses: The PRISMA statement. BMJ. (2009) 339:b2535–5. doi: 10.1136/bmj.b2535 21603045 PMC3090117

[35] Covidence Systematic Review Software. Melbourne, Australia: Veritas Health Innovation. (2025). Available online at: http://www.covidence.org./ (Accessed September 3, 2026).

[36] Hong QN Fàbregues S Bartlett G Boardman F Cargo M Dagenais P . The Mixed Methods Appraisal Tool (MMAT) version 2018 for information professionals and researchers. Educ Inf. (2018) 34:285–91. doi: 10.3233/EFI-180221

[37] Baig LA Tanzil S Shaikh S Hashmi I Khan MA Polkowski M . Effectiveness of training on de-escalation of violence and management of aggressive behavior faced by health care providers in a public sector hospital of Karachi. Pak J Med Sci. (2018) 34. doi: 10.12669/pjms.342.14432 29805396 PMC5954367

[38] Baldaçara L Ismael F Leite V Pereira LA Dos Santos RM Gomes Júnior VDP . Brazilian guidelines for the management of psychomotor agitation. Part 1. Non-pharmacological approach. Braz J Psychiatry. (2019) 41:153–67. doi: 10.1590/1516-4446-2018-0163 30540028 PMC6781680

[39] Brenig D Gade P Voellm B . Is mental health staff training in de-escalation techniques effective in reducing violent incidents in forensic psychiatric settings? – A systematic review of the literature. BMC Psychiatry. (2023) 23:246. doi: 10.1186/s12888-023-04714-y 37046228 PMC10099889

[40] Cahill D . The effect of ACT-SMART on nurses’ perceived level of confidence toward managing the aggressive and violent patient. Adv Emerg Nurs J. (2008) 30:252–68. doi: 10.1097/01.TME.0000334377.82646.0433079766

[41] Cowin L Davies R Estall G Berlin T Fitzgerald M Hoot S . De‐escalating aggression and violence in the mental health setting. Int J Ment Health Nurs. (2003) 12:64–73. doi: 10.1046/j.1440-0979.2003.00270.x 14685961

[42] Doreen Crawford DC . Equipping children’s nurses to de-escalate conflict and communicate challenging information Nursing Children and Young People (2020). Available online at: https://journals.rcni.com/nursing-children-and-young-people/cpd/equipping-childrens-nurses-to-deescalate-conflict-and-communicate-challenging-information-ncyp.2020.e1296/abs (Accessed February 10, 2026). 10.7748/ncyp.2020.e1296 32627510

[43] Dombrowsky TA . Responding to verbal abuse. Nurs (Lond). (2012) 42:58–61. doi: 10.1097/01.NURSE.0000421376.75122.4f 23076320

[44] Duncan G Schabbing M Gable BD . A novel simulation-based multidisciplinary verbal de-escalation training. Cureus. (2021) 13(12):e20849. doi: 10.7759/cureus.20849 35111487 PMC8794390

[45] Georgadarellis AG Baum CR . De-escalation techniques for the agitated pediatric patient. Pediatr Emerg Care. (2023) 39:535–9. doi: 10.1097/PEC.0000000000002989 37383009

[46] Homotoff K . Defusing aggressive behavior in the healthcare setting. JAAPA. (2021) 34:46–9. doi: 10.1097/01.JAA.0000758216.65209.27 34448778

[47] Mavandadi V Bieling PJ Madsen V . Effective ingredients of verbal de‐escalation: Validating an English modified version of the ‘De‐Escalating Aggressive Behaviour Scale’. J Psychiatr Ment Health Nurs. (2016) 23:357–68. doi: 10.1111/jpm.12310 27271938

[48] Moore N Ahmadpour N Brown M Poronnik P Davids J . Designing virtual reality–based conversational agents to train clinicians in verbal de-escalation skills: Exploratory usability study. JMIR Serious Games. (2022) 10:e38669. doi: 10.2196/38669 35793129 PMC9301562

[49] Pérez‐Toribio A Moreno‐Poyato AR Lluch‐Canut T Molina‐Martínez L Bastidas‐Salvadó A Puig‐Llobet M . Relationship between nurses’ use of verbal de‐escalation and mechanical restraint in acute inpatient mental health care: A retrospective study. Int J Ment Health Nurs. (2022) 31:339–47. doi: 10.1111/inm.12961 34837275

[50] Price O Baker J . Key components of de‐escalation techniques: A thematic synthesis. Int J Ment Health Nurs. (2012) 21:310–9. doi: 10.1111/j.1447-0349.2011.00793.x 22340073

[51] Price O Papastavrou Brooks C Johnston I McPherson P Goodman H Grundy A . Development and evaluation of a de-escalation training intervention in adult acute and forensic units: the EDITION systematic review and feasibility trial. Health Technol Assess. (2024) 28:1–120. doi: 10.3310/FGGW6874 38343036 PMC11017147

[52] Richter D . Nonphysical conflict management and deescalation, in: Violence in Mental Health Settings (2006). New York, NY: Springer New York (Accessed February 10, 2026). doi: 10.1007/978-0-387-33965-8_7

[53] Lafta RK Mousawi AMA . Violence against junior medical doctors in Iraq: A trial for de-escalation. J Emerg Med Trauma Acute Care. (2024) 2024. doi: 10.5339/jemtac.2024.cism.1

[54] See J Van Deusen R Claxton R Shenai N Rothenberger SD Donovan AK . CALMER conflict: A novel curriculum for graduating medical students to manage and defuse patient-provider conflict. J Gen Intern Med. (2025) 40:253–7. doi: 10.1007/s11606-024-08975-5 39103602 PMC11780236

[55] Moir C Baby M . Managing violence and aggression: Graduate-entry nursing students’ responses to pre-emptive communication skills education. Kaitiaki Nurs Res. (2022) 13:9–18. doi: 10.3316/informit.932444112644759

[56] Power T Kennedy P Chen H Martinez-Maldonado R McGregor C Johnson A . Learning to manage de-escalation through simulation: An exploratory study. Clin Simul Nurs. (2023) 77:23–9. doi: 10.1016/j.ecns.2023.01.00642574925

[57] Shenai N Fulmer V Gowl C See J Peterson R Van Deusen R . A verbal de-escalation standardized patient workshop for third- and fourth-year medical students. MedEdPORTAL. (2024) 11417. doi: 10.15766/mep_2374-8265.11417 39035103 PMC11258212

[58] Adams J Knowles A Irons G Roddy A Ashworth J . Assessing the effectiveness of clinical education to reduce the frequency and recurrence of workplace violence. Aust J Adv Nurs. (2017) 34:6–15. doi: 10.3316/informit.946627915561528

[59] Celofiga A Kores Plesnicar B Koprivsek J Moskon M Benkovic D Gregoric Kumperscak H . Effectiveness of de-escalation in reducing aggression and coercion in acute psychiatric units. A cluster randomized study. Front Psychiatry. (2022) 13:856153. doi: 10.3389/fpsyt.2022.856153 35463507 PMC9021532

[60] Feinstein RE . Violence prevention education program for psychiatric outpatient departments. Acad Psychiatry. (2014) 38:639–46. doi: 10.1007/s40596-014-0160-5 25059536

[61] Kronish A Alanko D Quinn VR Wulff C Stone E Wing R . De-escalation of the agitated pediatric patient: A standardized patient case for pediatric residents. MedEdPORTAL. (2024) 11388. doi: 10.15766/mep_2374-8265.11388 38463716 PMC10920402

[62] Arturén H Zetherström J Sjöström N Abrams D Johansson L . Handling conflict situations in psychosis inpatient care: Nursing staff experiences of the Interactive Approach model. J Psychiatr Ment Health Nurs. (2024) 31:1083–92. doi: 10.1111/jpm.13066 38796785

[63] Bowllan NM O’brien HP Blackwood C Cross WF Walsh P . Implementation and evaluation of a high-fidelity, interprofessional simulation project using standardized patients to address aggression in a psychiatric emergency department. J Am Psychiatr Nurses Assoc. (2025) 31:121–7. doi: 10.1177/10783903241308529 39772787

[64] Duncan G Schabbing MB Gable B . Multidisciplinary simulation-based verbal de-escalation training improves staff knowledge and clinical environment in an urban emergency department. Acad Psychiatry. (2024) 48:603–7. doi: 10.1007/s40596-024-02051-0 39317825

[65] Georgadarellis AG Shabanova V Hoffman P Tiyyagura G Auerbach M Langhan ML . Don’t get MAD: Managing agitation with de-escalation training in a pediatric emergency department. Pediatr Emerg Care. (2025) 42(3):186–92. doi: 10.1097/PEC.0000000000003526 41422423

[66] Phipps K . The utilization of empathy and self-awareness models to deescalate violence in the ambulatory clinic setting. J Patient Exp. (2024) 11:23743735241272261. doi: 10.1177/23743735241272261 39372660 PMC11450709

[67] Carreras Tartak J Rodriguez G Schwid M Meeker M Thomas M Roy H . Evaluating the feasibility and effectiveness of an interdisciplinary verbal de-escalation and implicit bias check training for agitation management in the emergency department. Risk Manag Healthc Policy. (2025) 18:1355–66. doi: 10.2147/RMHP.S513026 40264505 PMC12012381

[68] Thompson L Conklin A Holcomb B Moore C Staton L Zhang X . Verbal deescalation training: An initiative to increase confidence in the newly graduated nurse. J Nurses Prof Dev. (2025) 41:17–21. doi: 10.1097/NND.0000000000001104 39729350

[69] Somani R Muntaner C Hillan E Velonis AJ Smith P . A systematic review: Effectiveness of interventions to de-escalate workplace violence against nurses in healthcare settings. Saf Health Work. (2021) 12:289–95. doi: 10.1016/j.shaw.2021.04.004 34527388 PMC8430427

[70] Levin PF Hewitt JB Misner ST . Insights of nurses about assault in hospital‐based emergency departments. Image J Nurs Sch. (1998) 30:249–54. doi: 10.1111/j.1547-5069.1998.tb01300.x 9753840

[71] Honneth A . The Struggle for Recognition: The Moral Grammar of Social Conflicts. 1st MIT Press ed. Cambridge, Mass: MIT Press (1996). p. 215. Studies in contemporary German social thought.

[72] Hennein R Tiako MJN Bonumwezi J Tineo P Boatright D Crusto C . Vicarious racism, direct racism, and mental health among racialized minority healthcare workers. J Racial Ethn Health Disparities. (2025) 12:8–21. doi: 10.1007/s40615-023-01844-7 37935947

[73] Geoffrion S Hills DJ Ross HM Pich J Hill AT Dalsbø TK . Education and training for preventing and minimizing workplace aggression directed toward healthcare workers. Cochrane Database Syst Rev. (2020) 2020. doi: 10.1002/14651858.CD011860.pub2 32898304 PMC8094156

[74] Almulla O Tawash E Alaradi M Nair B . Building clinical confidence, the impact of de-escalation training on nurses’ management of aggression: An integrative review. Int Emerg Nurs. (2026) 85:101745. doi: 10.1016/j.ienj.2026.101745 41558135

[75] Vandecasteele R Robijn L Willems S De Maesschalck S Stevens PAJ . Barriers and facilitators to culturally sensitive care in general practice: A reflexive thematic analysis. BMC Prim Care. (2024) 25:381. doi: 10.1186/s12875-024-02630-y 39443846 PMC11515484

[76] Suárez Rojas RD Hashimoto D Sabbath EL . The mental states of aggressors: A biopsychosocial analysis of workplace violence reports in hospitals. Am J Ind Med. (2026) 69:132–43. doi: 10.1002/ajim.70047 41451695 PMC12803624

[77] Workplace Violence Prevention – Training: Tip Sheet. Available online at: https://ona.org/wp-content/uploads/2024/10/HS_Toolkit_TipSheetWorkplaceViolencePreventionTraining_20241029.pdf (Accessed February 10, 2026).

[78] Schulz-Quach C Gorla J Reynolds C Sethi R Lyver B . The life cycle of a code white: A framework for managing workplace violence. NEJM Catal. (2024) 5. doi: 10.1056/CAT.23.0352

[79] Carayon P Wooldridge A Hoonakker P Hundt AS Kelly MM . SEIPS 3.0: Human-centered design of the patient journey for patient safety. Appl Ergon. (2020) 84:103033. doi: 10.1016/j.apergo.2019.103033 31987516 PMC7152782

