# Supplementary material for: Verbal de-escalation techniques for the prevention and management of workplace violence in healthcare: a systematic review
Source: Front Psychiatry. 2026 Oct 14;17:1819502. doi: 10.3389/fpsyt.2026.1819502 (PMC13713309; doi:10.3389/fpsyt.2026.1819502)
Supplement: Supplementary file 1 [file DataSheet1.docx]

**Supplementary File 1.** Full electronic database search strategies.

Search Documentation

# Searches run August 29, 2024 in:

- Ovid MEDLINE(R) ALL 1946 to August 27, 2024
- Ovid Embase Classic+Embase 1947 to 2024 August 28
- Ovid Emcare Nursing 1995 to Present
- Ovid APA PsycInfo 1806 to August 2024 Week 4
- EBSCOhost Research Databases CINAHL Ultimate

# Update searches run January 30, 2026 in:

- Ovid MEDLINE(R) ALL 1946 to January 29, 2026
- Ovid Embase Classic+Embase 1947 to 2026 January 28
- Ovid Emcare Nursing 1995 to Present
- Ovid APA PsycInfo 1806 to January 2026 Week 5
- EBSCOhost Research Databases CINAHL Ultimate

# Search Strategies August 29, 2024:

## **Ovid MEDLINE(R) ALL 1946 to August 27, 2024**

Search Strategy:

| **#** | **Searches** | **Results** |
| --- | --- | --- |
| 1 | exp Violence/pc | 21212 |
| 2 | exp Violence/ and (de-escalat* or prevent* or mitigat* or difus* or defus* or redirect* or distract* or decreas* or reduc* or respon* or manag*).ti,kf,hw. | 15689 |
| 3 | ((de-escalat* or deescalat*) and (agitat* or agress* or violen* or behavio?r*)).mp. | 604 |
| 4 | (mitigat* adj4 (agitat* or agress* or violen* or behavio?r*)).mp. | 1979 |
| 5 | (difus* adj4 (agitat* or agress* or violen* or behavio?r*)).mp. | 0 |
| 6 | (defus* adj4 (agitat* or agress* or violen* or behavio?r*)).mp. | 34 |
| 7 | (redirect* adj4 (agitat* or agress* or violen* or behavio?r*)).mp. | 115 |
| 8 | (distract* adj4 (agitat* or agress* or violen* or behavio?r*)).mp. | 898 |
| 9 | (decreas* adj4 (agitat* or agress* or violen* or behavio?r*)).mp. | 14139 |
| 10 | (reduc* adj4 (agitat* or agress* or violen* or behavio?r*)).mp. | 48632 |
| 11 | (less* adj4 (agitat* or agress* or violen* or behavio?r*)).mp. | 10797 |
| 12 | "behavio?r* respon*".mp. | 23853 |
| 13 | (behavio?r* adj4 optimi*).mp. | 2535 |
| 14 | (BERT and behavio?r*).mp. | 81 |
| 15 | (agitat* adj4 manag*).mp. | 922 |
| 16 | (agress* adj4 manag*).mp. | 3 |
| 17 | "code white".mp. | 17 |
| 18 | (violen* adj4 manag*).mp. | 1174 |
| 19 | (agress* adj4 prevent*).mp. | 6 |
| 20 | (agitat* adj4 prevent*).mp. | 236 |
| 21 | (violen* adj4 prevent*).mp. | 6263 |
| 22 | (agitat* adj4 reduc*).mp. | 869 |
| 23 | (agress* adj4 reduc*).mp. | 3 |
| 24 | (violen* adj4 reduc*).mp. | 3159 |
| 25 | (restraint* adj6 (prevent* or reduc* or lessen* or decreas* or minimiz*)).mp. | 3075 |
| 26 | or/1-25 | 133654 |
| 27 | verbal*.mp. | 105155 |
| 28 | (non-coercive or noncoercive).mp. | 211 |
| 29 | (communicat* adj4 (de-escalat* or deescalat* or mitigat* or difus* or defus*)).mp. | 430 |
| 30 | (dialog* adj4 (de-escalat* or deescalat* or mitigat* or difus* or defus*)).mp. | 12 |
| 31 | (communicat* adj4 (manag* or respon* or prevent* or reduc*)).mp. | 17080 |
| 32 | (dialog* adj4 (manag* or respon* or prevent* or reduc*)).mp. | 665 |
| 33 | or/27-32 | 122949 |
| 34 | 26 and 33 | 2722 |

## Ovid Embase Classic+Embase 1947 to 2024 August 28

Search Strategy:

| **#** | **Searches** | **Results** |
| --- | --- | --- |
| 1 | exp Violence/pc | 8715 |
| 2 | exp Violence/ and (de-escalat* or prevent* or mitigat* or difus* or defus* or redirect* or distract* or decreas* or reduc* or respon* or manag*).ti,kf,hw. | 34770 |
| 3 | ((de-escalat* or deescalat*) and (agitat* or agress* or violen* or behavio?r*)).mp. | 982 |
| 4 | (mitigat* adj4 (agitat* or agress* or violen* or behavio?r*)).mp. | 2141 |
| 5 | (difus* adj4 (agitat* or agress* or violen* or behavio?r*)).mp. | 3 |
| 6 | (defus* adj4 (agitat* or agress* or violen* or behavio?r*)).mp. | 38 |
| 7 | (redirect* adj4 (agitat* or agress* or violen* or behavio?r*)).mp. | 156 |
| 8 | (distract* adj4 (agitat* or agress* or violen* or behavio?r*)).mp. | 1088 |
| 9 | (decreas* adj4 (agitat* or agress* or violen* or behavio?r*)).mp. | 17842 |
| 10 | (reduc* adj4 (agitat* or agress* or violen* or behavio?r*)).mp. | 42218 |
| 11 | (less* adj4 (agitat* or agress* or violen* or behavio?r*)).mp. | 13051 |
| 12 | "behavio?r* respon*".mp. | 27592 |
| 13 | (behavio?r* adj4 optimi*).mp. | 2888 |
| 14 | (BERT and behavio?r*).mp. | 100 |
| 15 | (agitat* adj4 manag*).mp. | 1491 |
| 16 | (agress* adj4 manag*).mp. | 50 |
| 17 | "code white".mp. | 33 |
| 18 | (violen* adj4 manag*).mp. | 1407 |
| 19 | (agress* adj4 prevent*).mp. | 22 |
| 20 | (agitat* adj4 prevent*).mp. | 356 |
| 21 | (violen* adj4 prevent*).mp. | 6885 |
| 22 | (agitat* adj4 reduc*).mp. | 1314 |
| 23 | (agress* adj4 reduc*).mp. | 18 |
| 24 | (violen* adj4 reduc*).mp. | 3505 |
| 25 | (restraint* adj6 (prevent* or reduc* or lessen* or decreas* or minimiz*)).mp. | 3781 |
| 26 | or/1-25 | 147301 |
| 27 | verbal*.mp. | 153400 |
| 28 | (non-coercive or noncoercive).mp. | 240 |
| 29 | (communicat* adj4 (de-escalat* or deescalat* or mitigat* or difus* or defus*)).mp. | 495 |
| 30 | (dialog* adj4 (de-escalat* or deescalat* or mitigat* or difus* or defus*)).mp. | 14 |
| 31 | (communicat* adj4 (manag* or respon* or prevent* or reduc*)).mp. | 22872 |
| 32 | (dialog* adj4 (manag* or respon* or prevent* or reduc*)).mp. | 765 |
| 33 | or/27-32 | 176836 |
| 34 | 26 and 33 | 3994 |

## Ovid Emcare Nursing 1995 to Present

Search Strategy:

| **#** | **Searches** | **Results** |
| --- | --- | --- |
| 1 | exp Violence/ and (de-escalat* or prevent* or mitigat* or difus* or defus* or redirect* or distract* or decreas* or reduc* or respon* or manag*).ti,kf,hw. | 18550 |
| 2 | ((de-escalat* or deescalat*) and (agitat* or agress* or violen* or behavio?r*)).mp. | 439 |
| 3 | (mitigat* adj4 (agitat* or agress* or violen* or behavio?r*)).mp. | 948 |
| 4 | (difus* adj4 (agitat* or agress* or violen* or behavio?r*)).mp. | 1 |
| 5 | (defus* adj4 (agitat* or agress* or violen* or behavio?r*)).mp. | 21 |
| 6 | (redirect* adj4 (agitat* or agress* or violen* or behavio?r*)).mp. | 53 |
| 7 | (distract* adj4 (agitat* or agress* or violen* or behavio?r*)).mp. | 439 |
| 8 | (decreas* adj4 (agitat* or agress* or violen* or behavio?r*)).mp. | 5849 |
| 9 | (reduc* adj4 (agitat* or agress* or violen* or behavio?r*)).mp. | 17226 |
| 10 | (less* adj4 (agitat* or agress* or violen* or behavio?r*)).mp. | 5975 |
| 11 | "behavio?r* respon*".mp. | 5149 |
| 12 | (behavio?r* adj4 optimi*).mp. | 854 |
| 13 | (BERT and behavio?r*).mp. | 29 |
| 14 | (agitat* adj4 manag*).mp. | 585 |
| 15 | (agress* adj4 manag*).mp. | 8 |
| 16 | "code white".mp. | 14 |
| 17 | (violen* adj4 manag*).mp. | 991 |
| 18 | (agress* adj4 prevent*).mp. | 4 |
| 19 | (agitat* adj4 prevent*).mp. | 148 |
| 20 | (violen* adj4 prevent*).mp. | 5419 |
| 21 | (agitat* adj4 reduc*).mp. | 550 |
| 22 | (agress* adj4 reduc*).mp. | 1 |
| 23 | (violen* adj4 reduc*).mp. | 2557 |
| 24 | (restraint* adj6 (prevent* or reduc* or lessen* or decreas* or minimiz*)).mp. | 1312 |
| 25 | or/1-24 | 55945 |
| 26 | verbal*.mp. | 50780 |
| 27 | (non-coercive or noncoercive).mp. | 153 |
| 28 | (communicat* adj4 (de-escalat* or deescalat* or mitigat* or difus* or defus*)).mp. | 235 |
| 29 | (dialog* adj4 (de-escalat* or deescalat* or mitigat* or difus* or defus*)).mp. | 7 |
| 30 | (communicat* adj4 (manag* or respon* or prevent* or reduc*)).mp. | 10319 |
| 31 | (dialog* adj4 (manag* or respon* or prevent* or reduc*)).mp. | 433 |
| 32 | or/26-31 | 61499 |
| 33 | 25 and 32 | 2106 |

## Ovid APA PsycInfo 1806 to August 2024 Week 4

Search Strategy:

| **#** | **Searches** | **Results** |
| --- | --- | --- |
| 1 | exp Violence/ and (de-escalat* or prevent* or mitigat* or difus* or defus* or redirect* or distract* or decreas* or reduc* or respon* or manag*).ti,id,hw. | 18661 |
| 2 | ((de-escalat* or deescalat*) and (agitat* or agress* or violen* or behavio?r*)).mp. | 656 |
| 3 | (mitigat* adj4 (agitat* or agress* or violen* or behavio?r*)).mp. | 1387 |
| 4 | (difus* adj4 (agitat* or agress* or violen* or behavio?r*)).mp. | 2 |
| 5 | (defus* adj4 (agitat* or agress* or violen* or behavio?r*)).mp. | 60 |
| 6 | (redirect* adj4 (agitat* or agress* or violen* or behavio?r*)).mp. | 220 |
| 7 | (distract* adj4 (agitat* or agress* or violen* or behavio?r*)).mp. | 1138 |
| 8 | (decreas* adj4 (agitat* or agress* or violen* or behavio?r*)).mp. | 12478 |
| 9 | (reduc* adj4 (agitat* or agress* or violen* or behavio?r*)).mp. | 30511 |
| 10 | (less* adj4 (agitat* or agress* or violen* or behavio?r*)).mp. | 11713 |
| 11 | "behavio?r* respon*".mp. | 16968 |
| 12 | (behavio?r* adj4 optimi*).mp. | 1287 |
| 13 | (BERT and behavio?r*).mp. | 164 |
| 14 | (agitat* adj4 manag*).mp. | 440 |
| 15 | (agress* adj4 manag*).mp. | 1 |
| 16 | "code white".mp. | 4 |
| 17 | (violen* adj4 manag*).mp. | 1499 |
| 18 | (agress* adj4 prevent*).mp. | 3 |
| 19 | (agitat* adj4 prevent*).mp. | 52 |
| 20 | (violen* adj4 prevent*).mp. | 7511 |
| 21 | (agitat* adj4 reduc*).mp. | 429 |
| 22 | (agress* adj4 reduc*).mp. | 6 |
| 23 | (violen* adj4 reduc*).mp. | 3730 |
| 24 | (restraint* adj6 (prevent* or reduc* or lessen* or decreas* or minimiz*)).mp. | 1511 |
| 25 | or/1-24 | 94343 |
| 26 | verbal*.mp. | 164182 |
| 27 | (non-coercive or noncoercive).mp. | 373 |
| 28 | (communicat* adj4 (de-escalat* or deescalat* or mitigat* or difus* or defus*)).mp. | 247 |
| 29 | (dialog* adj4 (de-escalat* or deescalat* or mitigat* or difus* or defus*)).mp. | 10 |
| 30 | (communicat* adj4 (manag* or respon* or prevent* or reduc*)).mp. | 12448 |
| 31 | (dialog* adj4 (manag* or respon* or prevent* or reduc*)).mp. | 928 |
| 32 | or/26-31 | 177369 |
| 33 | 25 and 32 | 3284 |

## EBSCOhost Research Databases CINAHL Ultimate

Thu, August 29, 2024 5:02:13 p.m.

Expanders - Apply equivalent subjects
Search modes - Proximity

| **#** | **Query** | **Results** |
| --- | --- | --- |
| S33 | S26 AND S32 | 2,031 |
| S32 | S27 OR S28 OR S29 OR S30 OR S31 | 45,542 |
| S31 | (dialog* N4 (manag* or respon* or prevent* or reduc*)) | 472 |
| S30 | (communicat* N4 (manag* or respon* or prevent* or reduc*)) | 10,508 |
| S29 | (dialog* N4 (de-escalat* or deescalat* or mitigat* or difus* or defus*)) | 8 |
| S28 | (communicat* N4 (de-escalat* or deescalat* or mitigat* or difus* or defus*)) | 223 |
| S27 | verbal* or non-coercive or noncoercive | 34,732 |
| S26 | S1 OR S2 OR S3 OR S4 OR S5 OR S6 OR S7 OR S8 OR S9 OR S10 OR S11 OR S12 OR S13 OR S14 OR S15 OR S16 OR S17 OR S18 OR S19 OR S20 OR S21 OR S22 OR S23 OR S24 OR S25 | 54,387 |
| S25 | (restraint* N6 (prevent* or reduc* or lessen* or decreas* or minimiz*)) | 1,232 |
| S24 | violen* N4 reduc* | 2,463 |
| S23 | agress* N4 reduc* | 5 |
| S22 | agitat* N4 reduc* | 505 |
| S21 | violen* N4 prevent* | 14,087 |
| S20 | agitat* N4 prevent* | 599 |
| S19 | agress* N4 prevent* | 2 |
| S18 | violen* N4 manag* | 1,115 |
| S17 | "code white" | 18 |
| S16 | agress* N4 manag* | 2 |
| S15 | agitat* N4 manag* | 575 |
| S14 | BERT AND ( behaviour* or behavior* ) | 13 |
| S13 | optimi* N4 (behavior* or behaviour*) | 659 |
| S12 | "behavior* respon*" OR "behaviour* respon*" | 2,710 |
| S11 | (less* N4 (agitat* or agress* or violen* or behavior* or behaviour*)) | 4,775 |
| S10 | (reduc* N4 (agitat* or agress* or violen* or behavior* or behaviour?)) | 14,295 |
| S9 | (decreas* N4 (agitat* or agress* or violen* or behavior* or behaviour*)) | 4,741 |
| S8 | (distract* N4 (agitat* or agress* or violen* or behavior* or behaviour*)) | 359 |
| S7 | (redirect* N4 (agitat* or agress* or violen* or behavior* or behaviour*)) | 42 |
| S6 | (defus* N4 (agitat* or agress* or violen* or behavior* or behaviour*)) | 41 |
| S5 | (difus* N4 (agitat* or agress* or violen* or behavior* or behaviour*)) | 1 |
| S4 | (mitigat* N4 (agitat* or agress* or violen* or behavior* or behaviour*)) | 807 |
| S3 | ( (de-escalat* or deescalat*) ) AND ( (agitat* or agress* or violen* or behavior* or behaviour*) ) | 403 |
| S2 | (MH "Violence+") AND TI ( (de-escalat* or prevent* or mitigat* or difus* or defus* or redirect* or distract* or decreas* or reduc* or respon* or manag*) ) | 7,642 |
| S1 | (MH "Violence+") AND SU ( (de-escalat* or prevent* or mitigat* or difus* or defus* or redirect* or distract* or decreas* or reduc* or respon* or manag*) ) | 22,644 |

# Search Strategies January 30, 2026

## Ovid MEDLINE(R) ALL 1946 to January 29, 2026

Search Strategy:

| **#** | **Searches** | **Results** |
| --- | --- | --- |
| 1 | exp Violence/pc | 22174 |
| 2 | exp Violence/ and (de-escalat* or prevent* or mitigat* or difus* or defus* or redirect* or distract* or decreas* or reduc* or respon* or manag*).ti,kf,hw. | 16593 |
| 3 | ((de-escalat* or deescalat*) and (agitat* or agress* or violen* or behavio?r*)).mp. | 749 |
| 4 | (mitigat* adj4 (agitat* or agress* or violen* or behavio?r*)).mp. | 2821 |
| 5 | (difus* adj4 (agitat* or agress* or violen* or behavio?r*)).mp. | 0 |
| 6 | (defus* adj4 (agitat* or agress* or violen* or behavio?r*)).mp. | 35 |
| 7 | (redirect* adj4 (agitat* or agress* or violen* or behavio?r*)).mp. | 140 |
| 8 | (distract* adj4 (agitat* or agress* or violen* or behavio?r*)).mp. | 986 |
| 9 | (decreas* adj4 (agitat* or agress* or violen* or behavio?r*)).mp. | 15255 |
| 10 | (reduc* adj4 (agitat* or agress* or violen* or behavio?r*)).mp. | 53734 |
| 11 | (less* adj4 (agitat* or agress* or violen* or behavio?r*)).mp. | 11563 |
| 12 | "behavio?r* respon*".mp. | 25645 |
| 13 | (behavio?r* adj4 optimi*).mp. | 3198 |
| 14 | (BERT and behavio?r*).mp. | 137 |
| 15 | (agitat* adj4 manag*).mp. | 1050 |
| 16 | (agress* adj4 manag*).mp. | 3 |
| 17 | "code white".mp. | 18 |
| 18 | (violen* adj4 manag*).mp. | 1307 |
| 19 | (agress* adj4 prevent*).mp. | 6 |
| 20 | (agitat* adj4 prevent*).mp. | 266 |
| 21 | (violen* adj4 prevent*).mp. | 7042 |
| 22 | (agitat* adj4 reduc*).mp. | 989 |
| 23 | (agress* adj4 reduc*).mp. | 3 |
| 24 | (violen* adj4 reduc*).mp. | 3564 |
| 25 | (restraint* adj6 (prevent* or reduc* or lessen* or decreas* or minimiz*)).mp. | 3279 |
| 26 | or/1-25 | 145640 |
| 27 | verbal*.mp. | 111681 |
| 28 | (non-coercive or noncoercive).mp. | 239 |
| 29 | (communicat* adj4 (de-escalat* or deescalat* or mitigat* or difus* or defus*)).mp. | 584 |
| 30 | (dialog* adj4 (de-escalat* or deescalat* or mitigat* or difus* or defus*)).mp. | 16 |
| 31 | (communicat* adj4 (manag* or respon* or prevent* or reduc*)).mp. | 19902 |
| 32 | (dialog* adj4 (manag* or respon* or prevent* or reduc*)).mp. | 765 |
| 33 | or/27-32 | 132481 |
| 34 | 26 and 33 | 2977 |
| 35 | (2024082* or 202409* or 20241* or 2025* or 2026*).dt,ez,da. | 2569497 |
| 36 | 34 and 35 | 283 |

## Ovid Embase Classic+Embase 1947 to 2026 January 28

Search Strategy:

| **#** | **Searches** | **Results** |
| --- | --- | --- |
| 1 | exp Violence/pc | 18961 |
| 2 | exp Violence/ and (de-escalat* or prevent* or mitigat* or difus* or defus* or redirect* or distract* or decreas* or reduc* or respon* or manag*).ti,kf,hw. | 68846 |
| 3 | ((de-escalat* or deescalat*) and (agitat* or agress* or violen* or behavio?r*)).mp. | 1253 |
| 4 | (mitigat* adj4 (agitat* or agress* or violen* or behavio?r*)).mp. | 3108 |
| 5 | (difus* adj4 (agitat* or agress* or violen* or behavio?r*)).mp. | 0 |
| 6 | (defus* adj4 (agitat* or agress* or violen* or behavio?r*)).mp. | 40 |
| 7 | (redirect* adj4 (agitat* or agress* or violen* or behavio?r*)).mp. | 188 |
| 8 | (distract* adj4 (agitat* or agress* or violen* or behavio?r*)).mp. | 1247 |
| 9 | (decreas* adj4 (agitat* or agress* or violen* or behavio?r*)).mp. | 19784 |
| 10 | (reduc* adj4 (agitat* or agress* or violen* or behavio?r*)).mp. | 50266 |
| 11 | (less* adj4 (agitat* or agress* or violen* or behavio?r*)).mp. | 14192 |
| 12 | "behavio?r* respon*".mp. | 30069 |
| 13 | (behavio?r* adj4 optimi*).mp. | 3720 |
| 14 | (BERT and behavio?r*).mp. | 170 |
| 15 | (agitat* adj4 manag*).mp. | 1775 |
| 16 | (agress* adj4 manag*).mp. | 51 |
| 17 | "code white".mp. | 39 |
| 18 | (violen* adj4 manag*).mp. | 1582 |
| 19 | (agress* adj4 prevent*).mp. | 22 |
| 20 | (agitat* adj4 prevent*).mp. | 468 |
| 21 | (violen* adj4 prevent*).mp. | 7875 |
| 22 | (agitat* adj4 reduc*).mp. | 1625 |
| 23 | (agress* adj4 reduc*).mp. | 18 |
| 24 | (violen* adj4 reduc*).mp. | 4092 |
| 25 | (restraint* adj6 (prevent* or reduc* or lessen* or decreas* or minimiz*)).mp. | 4103 |
| 26 | or/1-25 | 200782 |
| 27 | verbal*.mp. | 172648 |
| 28 | (non-coercive or noncoercive).mp. | 275 |
| 29 | (communicat* adj4 (de-escalat* or deescalat* or mitigat* or difus* or defus*)).mp. | 680 |
| 30 | (dialog* adj4 (de-escalat* or deescalat* or mitigat* or difus* or defus*)).mp. | 16 |
| 31 | (communicat* adj4 (manag* or respon* or prevent* or reduc*)).mp. | 27167 |
| 32 | (dialog* adj4 (manag* or respon* or prevent* or reduc*)).mp. | 894 |
| 33 | or/27-32 | 200480 |
| 34 | 26 and 33 | 5168 |
| 35 | limit 34 to dc=20240827-20260130 | 875 |

## Ovid Emcare Nursing 1995 to Present

Search Strategy:

| **#** | **Searches** | **Results** |
| --- | --- | --- |
| 1 | exp Violence/ and (de-escalat* or prevent* or mitigat* or difus* or defus* or redirect* or distract* or decreas* or reduc* or respon* or manag*).ti,kf,hw. | 40104 |
| 2 | ((de-escalat* or deescalat*) and (agitat* or agress* or violen* or behavio?r*)).mp. | 578 |
| 3 | (mitigat* adj4 (agitat* or agress* or violen* or behavio?r*)).mp. | 1321 |
| 4 | (difus* adj4 (agitat* or agress* or violen* or behavio?r*)).mp. | 1 |
| 5 | (defus* adj4 (agitat* or agress* or violen* or behavio?r*)).mp. | 22 |
| 6 | (redirect* adj4 (agitat* or agress* or violen* or behavio?r*)).mp. | 59 |
| 7 | (distract* adj4 (agitat* or agress* or violen* or behavio?r*)).mp. | 479 |
| 8 | (decreas* adj4 (agitat* or agress* or violen* or behavio?r*)).mp. | 6349 |
| 9 | (reduc* adj4 (agitat* or agress* or violen* or behavio?r*)).mp. | 19596 |
| 10 | (less* adj4 (agitat* or agress* or violen* or behavio?r*)).mp. | 6411 |
| 11 | "behavio?r* respon*".mp. | 5665 |
| 12 | (behavio?r* adj4 optimi*).mp. | 1061 |
| 13 | (BERT and behavio?r*).mp. | 64 |
| 14 | (agitat* adj4 manag*).mp. | 666 |
| 15 | (agress* adj4 manag*).mp. | 8 |
| 16 | "code white".mp. | 19 |
| 17 | (violen* adj4 manag*).mp. | 1124 |
| 18 | (agress* adj4 prevent*).mp. | 4 |
| 19 | (agitat* adj4 prevent*).mp. | 167 |
| 20 | (violen* adj4 prevent*).mp. | 6098 |
| 21 | (agitat* adj4 reduc*).mp. | 615 |
| 22 | (agress* adj4 reduc*).mp. | 1 |
| 23 | (violen* adj4 reduc*).mp. | 2909 |
| 24 | (restraint* adj6 (prevent* or reduc* or lessen* or decreas* or minimiz*)).mp. | 1437 |
| 25 | or/1-24 | 80789 |
| 26 | verbal*.mp. | 59694 |
| 27 | (non-coercive or noncoercive).mp. | 180 |
| 28 | (communicat* adj4 (de-escalat* or deescalat* or mitigat* or difus* or defus*)).mp. | 332 |
| 29 | (dialog* adj4 (de-escalat* or deescalat* or mitigat* or difus* or defus*)).mp. | 8 |
| 30 | (communicat* adj4 (manag* or respon* or prevent* or reduc*)).mp. | 12082 |
| 31 | (dialog* adj4 (manag* or respon* or prevent* or reduc*)).mp. | 494 |
| 32 | or/26-31 | 72256 |
| 33 | 25 and 32 | 2793 |
| 34 | limit 33 to dc=20240827-20260130 | 300 |

## Ovid APA PsycInfo 1806 to January 2026 Week 5

Search Strategy:

| **#** | **Searches** | **Results** |
| --- | --- | --- |
| 1 | exp Violence/ and (de-escalat* or prevent* or mitigat* or difus* or defus* or redirect* or distract* or decreas* or reduc* or respon* or manag*).ti,id,hw. | 19992 |
| 2 | ((de-escalat* or deescalat*) and (agitat* or agress* or violen* or behavio?r*)).mp. | 745 |
| 3 | (mitigat* adj4 (agitat* or agress* or violen* or behavio?r*)).mp. | 1749 |
| 4 | (difus* adj4 (agitat* or agress* or violen* or behavio?r*)).mp. | 2 |
| 5 | (defus* adj4 (agitat* or agress* or violen* or behavio?r*)).mp. | 61 |
| 6 | (redirect* adj4 (agitat* or agress* or violen* or behavio?r*)).mp. | 229 |
| 7 | (distract* adj4 (agitat* or agress* or violen* or behavio?r*)).mp. | 1211 |
| 8 | (decreas* adj4 (agitat* or agress* or violen* or behavio?r*)).mp. | 13117 |
| 9 | (reduc* adj4 (agitat* or agress* or violen* or behavio?r*)).mp. | 32710 |
| 10 | (less* adj4 (agitat* or agress* or violen* or behavio?r*)).mp. | 12337 |
| 11 | "behavio?r* respon*".mp. | 17822 |
| 12 | (behavio?r* adj4 optimi*).mp. | 1418 |
| 13 | (BERT and behavio?r*).mp. | 190 |
| 14 | (agitat* adj4 manag*).mp. | 472 |
| 15 | (agress* adj4 manag*).mp. | 1 |
| 16 | "code white".mp. | 4 |
| 17 | (violen* adj4 manag*).mp. | 1605 |
| 18 | (agress* adj4 prevent*).mp. | 3 |
| 19 | (agitat* adj4 prevent*).mp. | 54 |
| 20 | (violen* adj4 prevent*).mp. | 8322 |
| 21 | (agitat* adj4 reduc*).mp. | 463 |
| 22 | (agress* adj4 reduc*).mp. | 6 |
| 23 | (violen* adj4 reduc*).mp. | 4071 |
| 24 | (restraint* adj6 (prevent* or reduc* or lessen* or decreas* or minimiz*)).mp. | 1586 |
| 25 | or/1-24 | 100696 |
| 26 | verbal*.mp. | 170223 |
| 27 | (non-coercive or noncoercive).mp. | 395 |
| 28 | (communicat* adj4 (de-escalat* or deescalat* or mitigat* or difus* or defus*)).mp. | 301 |
| 29 | (dialog* adj4 (de-escalat* or deescalat* or mitigat* or difus* or defus*)).mp. | 12 |
| 30 | (communicat* adj4 (manag* or respon* or prevent* or reduc*)).mp. | 13454 |
| 31 | (dialog* adj4 (manag* or respon* or prevent* or reduc*)).mp. | 992 |
| 32 | or/26-31 | 184498 |
| 33 | 25 and 32 | 3474 |
| 34 | limit 33 to up=20240827-20260130 | 181 |

## EBSCOhost Research Databases CINAHL Ultimate

((MH "Violence+" OR MH "Violence+") AND SU ( (de-escalat* or prevent* or mitigat* or difus* or defus* or redirect* or distract* or decreas* or reduc* or respon* or manag*) ) OR ( (de-escalat* or deescalat*) ) AND ( (agitat* or agress* or violen* or behavior* or behaviour*) ) OR ((mitigat* OR difus* OR defus* OR redirect* OR distract* OR decrease* OR reduc* OR less*) N4 (agitat* or agress* or violen* or behavior* or behaviour*))) OR behavior* respon* OR behaviour* respon* OR optimi* behavior* OR optimi* behaviour* OR (BERT and (behaviour* OR behavior*)) OR ((agitat* OR agress* OR violen* OR restraint*) N4 (prevent* OR reduc* OR lessen* OR decreas* OR minimiz* OR manag*)) OR "code white") AND (verbal* OR non-coercive OR noncoercive OR ((communicat* OR dialog*) N4 (manag* OR respon* OR prevent* OR reduc* OR de-escalat* OR deescalat* OR mitigat* OR difus* OR defus*))) AND ((EM 20240827- OR (ZD "in process" AND RD 20240827-)))

211 results in database Jan 30 2026

Export all results requested 12:09 January 30 2026
