# Supplementary material for: Verbal de-escalation techniques for the prevention and management of workplace violence in healthcare: a systematic review
Source: Front Psychiatry. 2026 Oct 14;17:1819502. doi: 10.3389/fpsyt.2026.1819502 (PMC13713309; doi:10.3389/fpsyt.2026.1819502)
Supplement: Supplementary file 2 [file DataSheet2.docx]

**Supplementary File 2.** Extended theoretical and disciplinary framework mapping of verbal de-escalation techniques.

This supplementary document provides an extended theoretical and disciplinary mapping of verbal de-escalation techniques identified in the systematic review. The primary theoretical models and disciplinary fields presented in Table 2 reflect the most direct grounding reported in the included studies. This supplementary file expands on broader theoretical and interdisciplinary perspectives that may inform understanding and future research on verbal de-escalation in healthcare settings.

**#1 Technique-Specific Theoretical and Disciplinary Mapping**

**Overview**

This section provides an expanded mapping of additional theoretical models and disciplinary perspectives related to each verbal de-escalation technique presented in the Table 2. The primary theoretical models and disciplinary fields in the main manuscript reflect those most directly described in the included studies. The material below broadens the conceptual context by identifying additional theoretical and interdisciplinary perspectives that may inform interpretation, implementation, and future research.

The references listed in this supplementary file include foundational theoretical works and are not part of the systematic review evidence base.

Expanded Theoretical and Disciplinary Perspectives

**#1 Limit-Setting and Establishing Boundaries**

**Primary (in Main Table):**

- Behavioural and social learning models of contingency management and boundary-setting
- Therapeutic limit-setting within milieu therapy

**Other Relevant Theories:**

- Dialectical Behaviour Therapy (DBT) — validation with firm limits; acceptance and change
- Transactional Analysis — ego states and boundary clarity in interpersonal transactions
- Attachment theory — secure base concepts; protective caregiving boundaries
- Systems and family systems theory — boundary maintenance at individual and systemic levels
- Psychodynamic theory — object relations; containment; internal working models of safety
- Moral development and ethics of care (Gilligan) — responsibility frameworks
- Neurobiology of stress — how clear boundaries activate parasympathetic recovery

**Other Relevant Disciplines:**

- Social work and counselling ethics
- Forensic psychology and criminology
- Occupational therapy (therapeutic use of structure and routine)
- Organizational behaviour and management
- Education and child development
- Neuroscience and psychobiology
- Legal and policy studies (duty of care; patients' rights)

**#2 Empathetic Tone and Pace of Speech**

**Primary (in Main Table):**

- Client-centred (humanistic) communication theory and affect regulation
- Crisis intervention and de-escalation frameworks

**Other Relevant Theories:**

- Neuroscience of empathy and mirror neurons — emotional contagion mechanisms
- Polyvagal theory (Porges) — vagal tone and parasympathetic activation through voice
- Psychoacoustics and prosody — linguistic properties of vocal tone; paralinguistic cues
- Emotional intelligence models (Goleman) — emotional awareness and regulation
- Nonverbal communication theory (Mehrabian's vocal tone dominance; Birdwhistell's kinesics)
- Narrative therapy — therapeutic use of language and tone in reframing
- Motivational interviewing — spirit of MI conveyed through tone
- Trauma-informed care — tone as safety signal; co-regulation in recovery

**Other Relevant Disciplines:**

- Linguistics and sociolinguistics (prosody, code-switching, register)
- Speech pathology and voice therapy
- Music therapy (acoustic properties of human voice)
- Neuroscience and neurobiology
- Theatre and performance studies (voice training, emotional authenticity)
- Organizational communication and leadership
- Cultural psychology (cultural norms around tone and emotion)

**#3 Attentive Listening Approach**

**Primary (in Main Table):**

- Active listening and empathic communication models
- Person-centred counselling and therapeutic alliance frameworks

**Other Relevant Theories:**

- Social constructionism — meaning-making through dialogue; co-construction of understanding
- Cognitive load theory — reducing patient cognitive burden through full attention
- Hermeneutics — art of interpretation; bridging meaning gaps
- Phenomenological approaches — bracketing assumptions; lived experience of the other
- Dialogic ethics (Martin Buber) — I-Thou relationship; authentic encounter
- Symbolism and semiotics — understanding verbal and non-verbal symbols
- Cultural humility frameworks — listening as cultural bridge-building
- Mindfulness and acceptance-based approaches — present-moment, non-judgmental awareness
- Intersubjectivity theory — mutual recognition through attentive presence

**Other Relevant Disciplines:**

- Anthropology and ethnography (participant observation; cultural understanding)
- Philosophy (epistemology, ethics, phenomenology, hermeneutics)
- Literary studies and narrative analysis
- Sociology (interaction rituals, face-work)
- Speech and hearing science
- Mindfulness-based interventions and contemplative studies
- Organizational psychology and management (listening in teams)
- Feminist theory and ethics of care

**#4 Verbalizing Intentions to Collaboratively De-Escalate**

**Primary (in Main Table):**

- Collaborative problem-solving and shared decision-making models
- Relational and therapeutic alliance frameworks

**Other Relevant Theories:**

- Transparency and trust theory — explicit intent-signalling builds psychological safety
- Face-saving and impression management (Goffman) — maintaining dignity for all parties
- Game theory and cooperative agreements — signalling intent to cooperate
- Social contract theory — resetting relational contracts in conflict moments
- Expectancy violation theory — how violations of negative expectations shift outcomes
- Message framing theory — importance of how collaborative intent is framed
- Negotiation theory — interest-based negotiation; separating people from problems
- Recovery model in mental health — collaboration as cornerstone of person-centered care

**Other Relevant Disciplines:**

- Mediation and conflict resolution (professional practice)
- Law and legal studies (informed consent, agreements, duty of care)
- Organizational development and change management
- Political science and diplomacy
- Business and management studies
- Consumer studies and customer relations
- Community psychology and peacebuilding

**#5 Repeating Instructions and Concerns**

**Primary (in Main Table):**

- Cognitive-behavioural and information-processing models
- Health communication theory (message clarity, redundancy, accessibility)

**Other Relevant Theories:**

- Cognitive psychology of attention and memory — working memory constraints; spaced repetition
- Schema theory — activating relevant cognitive frameworks through repetition
- Reinforcement schedules — partial reinforcement and repetition in behaviour modification
- Accessibility and readability theory — plain language; simplification for comprehension
- Dual coding theory — combining verbal repetition with non-verbal cues for retention
- Communication disorders and aphasia — adaptations for processing difficulties
- Stress and cognitive narrowing — how acute stress reduces cognitive capacity
- Pragmatic linguistics — conversational repair; explicit signalling when misunderstanding may occur
- Information design — principles of effective message design and repeated messaging

**Other Relevant Disciplines:**

- Cognitive science and experimental psychology
- Neurolinguistics and aphasiology
- Health literacy and patient education
- Instructional design and learning sciences
- Linguistics and language disorders (Speech-Language Pathology)
- UX and information design
- Public health communication
- Adult education and training

**#6 Developing Open Communication Channels**

**Primary (in Main Table):**

- Patient-centred communication
- Therapeutic alliance and relational frameworks
- Motivational interviewing-influenced models

**Other Relevant Theories:**

- Transparency and psychological safety (Edmondson) — creating environments where people feel safe to speak
- Social support theory — communication as instrumental and emotional support vehicle
- Reciprocal self-disclosure — vulnerability and authenticity building connection
- Narrative identity theory — allowing patients to tell their stories; co-constructing identity
- Relational frame theory — symbolic relations and meaning in language
- Positive psychology and strengths-based approaches — focusing on what's working
- Feminist standpoint theory — centering marginalized voices; power dynamics
- Community of practice — shared understanding through collective dialogue
- Organizational learning — creating cultures of openness and feedback

**Other Relevant Disciplines:**

- Interpersonal and relational psychology
- Group dynamics and small group communication
- Organizational culture and leadership studies
- Gender studies and feminist theory
- Social work and casework practice
- Education (student-centered learning; dialogic pedagogy)
- Community development and participatory action research
- Journalism and storytelling

**#7 Appropriate Negotiation Tactics**

**Primary (in Main Table):**

- Conflict resolution and negotiation theory
- Procedural justice models
- Collaborative problem-solving frameworks

**Other Relevant Theories:**

- Interest-based relational (IBR) negotiation (Fisher & Ury) — separating positions from interests
- Game theory and mutual gains — zero-sum vs. win-win framing
- Social exchange theory — reciprocity and mutual benefit in negotiated agreements
- Distributive vs. integrative negotiation — claiming and creating value
- Power dynamics and critical theory — acknowledging healthcare relationship asymmetries
- Fairness heuristics — perception of procedural justice affecting compliance
- Emotion regulation in negotiation — managing emotions to maintain problem-solving focus
- Mediation and facilitation theory — third-party intervention and shuttle diplomacy
- Organizational justice — distributive, procedural, and interactional justice in healthcare

**Other Relevant Disciplines:**

- Law and legal negotiation (arbitration, mediation practice)
- Organizational conflict management
- Industrial relations and labour studies
- Political science and international relations
- Business and commerce (sales, contracts, mergers)
- Diplomacy and peacebuilding
- Environmental dispute resolution
- Consumer protection and patient advocacy

**#8 Offering Treatment Choices or Amenities**

**Primary (in Main Table):**

- Behavioural reinforcement models
- Autonomy-supportive care models
- Self-determination theory (choice and control)

**Other Relevant Theories:**

- Operant conditioning and positive reinforcement — behavioural shaping through incentives
- Environmental psychology — impact of environmental modifications on affect
- Person-environment fit theory — matching needs with environmental affordances
- Recovery model — personal agency and choice as therapeutic mechanisms
- Stigma reduction — offering normal amenities as anti-stigma practice
- Dignity and person-centeredness — respecting preferences and autonomy as ethical imperatives
- Behavioural momentum — building cooperation through small, agreed-upon steps
- Sensory integration theory — calming sensory experiences reducing arousal
- Quality of life frameworks — wellbeing beyond medical outcomes; holistic care

**Other Relevant Disciplines:**

- Interior design and environmental design
- Consumer psychology and behaviour
- Nutritional science and food studies
- Recreation and leisure studies
- Disability studies (accommodation, universal design)
- Ethics and bioethics
- Occupational therapy
- User experience (UX) design

**#9 Offering Cool Down Periods or Relocating the Patient**

**Primary (in Main Table):**

- Arousal reduction and emotion-regulation models
- Behavioural time-out frameworks
- Environmental stress theories

**Other Relevant Theories:**

- Neurobiology of stress response — amygdala hijacking; parasympathetic recovery; window of tolerance
- Allostatic load — how cumulative stressors tax regulatory systems; recovery necessity
- Environmental psychology and restorative environments — nature, space, light, quiet reducing arousal
- Cognitive reappraisal — time and distance allowing re-evaluation and cognitive flexibility
- Temporal dynamics of emotion — emotion decay curves; natural affect trajectories
- Safety planning and trauma-informed care — predictable, safe spaces as healing modalities
- Sensory processing sensitivity — individual differences in arousal; customized de-arousal
- Environmental stressors (noise, crowding, temperature) — removing or modifying triggers
- Attention restoration theory — cognitive recovery through natural/quiet environments

**Other Relevant Disciplines:**

- Neuroscience and psychobiology (stress physiology)
- Environmental design and architecture
- Landscape architecture and nature-based interventions
- Mindfulness and meditation studies
- Trauma psychology and trauma-informed care
- Environmental health
- Facilities management and workplace design
- Recreation therapy and nature therapy

**#10 Debriefing After the Escalated Incident**

**Primary (in Main Table):**

- Critical incident stress debriefing (CISD) and reflective practice
- Learning-from-events and quality improvement theory

**Other Relevant Theories:**

- Reflective practice (Schön) — double-loop learning; theory-in-practice reflection
- Post-traumatic growth — meaning-making after difficult events; transformative learning
- Narrative therapy and re-authoring — constructing new meanings around past events
- Psychological debriefing and processing — emotional processing; preventing PTSD
- Situated learning theory — learning embedded in social context; communities of practice
- Error analysis and safety science — root cause analysis; systems thinking in healthcare
- Trauma-informed debriefing — avoiding re-traumatization; supportive approaches
- Organizational learning and knowledge management — capturing and disseminating lessons
- Accountability and restorative justice — repairing harm; rebuilding trust

**Other Relevant Disciplines:**

- Adult education and lifelong learning
- Organizational development and change management
- Quality improvement and patient safety (Lean, Six Sigma)
- Social work and case management (aftercare)
- Criminal justice and restorative justice programs
- Trauma counselling and psychotherapy
- Nursing and health professions education
- Systems engineering and human factors
- Occupational health and workplace safety

**#2 Cross-Cutting Theoretical Frameworks**

**Overview**

The frameworks outlined below represent broader conceptual models that may inform multiple verbal de-escalation techniques identified in this review. While not consistently or explicitly reported across the included studies, these models provide interdisciplinary perspectives that may support interpretation, implementation, and future research related to de-escalation practices in healthcare settings.

Implementation Science & Behaviour Change

- **Theoretical Domains Framework (TDF)** — 14 domains of behaviour change applicable to staff adoption of de-escalation
- **Capabilities, Opportunities, Motivations (COM-B)** — model of behaviour change applicable to staff and patient behaviour
- **Consolidated Framework for Implementation Research (CFIR)** — guides implementation research design
- **Normalization Process Theory** — how new practices become normalized in healthcare settings

Trauma-Informed Care

- **Trauma-informed principles** — safety, trustworthiness, choice, collaboration, empowerment applicable across all techniques
- **Polyvagal theory and window of tolerance** — neurobiological readiness for learning and interaction
- **Complex trauma and attachment** — how early trauma shapes de-escalation needs and responses

Person-Centred and Recovery Models

- **Recovery-focused mental health care** — de-escalation as supporting agency and autonomy
- **Social determinants of health** — contextualizing aggression/agitation within broader social, economic, environmental factors
- **Intersectionality** — how identity, power, and systemic oppression intersect with de-escalation

Systems and Complexity Theory

- **Organizational culture and climate** — how institutional factors enable or inhibit de-escalation
- **Human factors and ergonomics** — designing systems, workflows, environments to support de-escalation
- **Network theory** — de-escalation embedded in relational networks of staff, patients, families
- **Ecological systems theory** — multiple levels of influence (individual, interpersonal, organizational, societal)

Ethical Frameworks

- **Bioethics** — autonomy, beneficence, justice, non-maleficence as undergirding principles
- **Care ethics** — relationships, interdependence, meeting concrete needs
- **Virtue ethics** — character development in healthcare professionals (compassion, practical wisdom)
- **Justice frameworks** — equity in access to de-escalation; recognizing power asymmetries

**#3 Disciplinary Ecosystem Informing Verbal De-Escalation**

**Overview**

This section outlines additional disciplinary perspectives that may inform conceptual understanding of verbal de-escalation in healthcare. These disciplines are not necessarily represented in the included studies, but reflect broader intellectual fields relevant to the techniques identified in this review.

| **Discipline** | **Contribution Areas** |
| --- | --- |
| **Neuroscience & Neurobiology** | Physiological basis of arousal, emotion regulation, stress response, mirror neurons, vagal tone, neuroplasticity |
| **Linguistics & Sociolinguistics** | Prosody, semantics, pragmatics, discourse analysis, code-switching, language development, aphasia |
| **Anthropology** | Cultural norms around emotion, conflict, authority; ethnographic understanding of diverse populations |
| **Philosophy** | Epistemology, ethics, phenomenology, hermeneutics, dialogue theory |
| **Sociology** | Interaction rituals, status and power dynamics, organizational culture, stigma, medicalization |
| **Social Work** | Casework, family systems, community development, ethical practice, vulnerability |
| **Theatre & Performance** | Authenticity, presence, voice training, emotional truth, audience-performer dynamics |
| **Environmental Design & Architecture** | Therapeutic spaces, sensory environments, safety design, accessibility |
| **Occupational Therapy** | Meaningful activity, environmental modification, therapeutic use of self, holistic health |
| **Music Therapy** | Acoustic properties, rhythm, emotional resonance, non-verbal communication |
| **Education & Learning Sciences** | Cognitive load, instructional design, adult learning, reflective practice, pedagogy |
| **Organizational Development** | Organizational change, culture, leadership, team dynamics, implementation science |
| **Conflict Resolution & Mediation (Professional Field)** | Mediation practice, facilitation, interest-based negotiation, peace-building |
| **Law & Legal Studies** | Informed consent, duty of care, patient rights, liability, restorative justice |
| **Gender Studies & Feminist Theory** | Power relations, voice, standpoint theory, care ethics, lived experience of marginalization |
| **Disability Studies** | Universal design, accommodation, autonomy, social models of disability |
| **Quality Improvement & Patient Safety** | Lean, Six Sigma, error analysis, systems thinking, root cause analysis |
| **Public Health** | Population approaches, health messaging, health literacy, epidemiology of violence |
| **Political Science & Diplomacy** | Negotiation at scale, international relations, power dynamics, consensus-building |
| **Business & Management** | Leadership, communication, organizational behaviour, change management, customer relations |

**#4 Key Foundational References**

**Overview**

The references listed below represent foundational theoretical works relevant to the conceptual frameworks discussed in this supplementary file. These sources provide background context and are not part of the systematic review evidence base.

**Self-Determination Theory (choice, autonomy, patient engagement)**

- Deci EL, Ryan RM. The "what" and "why" of goal pursuits: Human needs and the self-determination of behavior. Psychological Inquiry. 2000;11(4):227-268.

- Ryan RM, Patrick H, Deci EL, Williams GC. Facilitating health behaviour change and its maintenance: Interventions based on self-determination theory. European Health Psychologist. 2008;10:2-5.

- Ng JY, Ntoumanis N, Thøgersen-Ntoumani C, et al. Self-determination theory applied to health contexts: A meta-analysis. Perspectives on Psychological Science. 2012;7(4):325-340.

**Polyvagal Theory (arousal, tone of voice, cool-down, safety signalling)**

- Porges SW. The polyvagal theory: New insights into adaptive reactions of the autonomic nervous system. Cleveland Clinic Journal of Medicine. 2009;76(Suppl 2):S86-S90.

- Porges SW. Polyvagal theory: A science of safety. Frontiers in Integrative Neuroscience. 2022;16:871227.

- Beauchaine TP, Gatzke-Kopp L, Mead HK. Polyvagal theory and developmental psychopathology: Emotion dysregulation and conduct problems from preschool to adolescence. Biological Psychology. 2007;74(2):174-184.

**Therapeutic Alliance / Working Alliance (collaboration, rapport, attentive listening)**

- Bordin ES. The generalizability of the psychoanalytic concept of the working alliance. Psychotherapy: Theory, Research & Practice. 1979;16(3):252-260.

- Horvath AO, Symonds BD. Relation between working alliance and outcome in psychotherapy: A meta-analysis. Journal of Counseling Psychology. 1991;38(2):139-149.

**Client-Centred / Person-Centred Therapy (empathic tone, attentive listening)**

- Rogers CR. Client-Centered Therapy: Its Current Practice, Implications, and Theory. Boston: Houghton Mifflin; 1951.

- Rogers CR. The necessary and sufficient conditions of therapeutic personality change. Journal of Consulting Psychology. 1957;21(2):95-103.

**Motivational Interviewing (open communication, collaboration, autonomy support)**

- Miller WR, Rollnick S. Motivational Interviewing: Helping People Change (3rd ed.). New York: Guilford Press; 2013.

- Markland D, Ryan RM, Tobin VJ, Rollnick S. Motivational interviewing and self-determination theory. Journal of Social and Clinical Psychology. 2005;24(6):811-831.

**Negotiation and Conflict Resolution Theory (negotiation tactics, procedural justice)**

- Fisher R, Ury W, Patton B. Getting to Yes: Negotiating Agreement Without Giving In (3rd ed.). New York: Penguin; 2011.

- Deutsch M, Coleman PT, Marcus EC (eds). The Handbook of Conflict Resolution: Theory and Practice (3rd ed.). San Francisco: Jossey-Bass; 2014.

**Critical Incident Stress Debriefing / Reflective Practice (debriefing)**

- Mitchell JT. When disaster strikes: The critical incident stress debriefing process. Journal of Emergency Medical Services. 1983;8(1):36-39.

- Schön DA. The Reflective Practitioner: How Professionals Think in Action. New York: Basic Books; 1983.

**Cognitive-Behavioural Models (repeating instructions, information processing)**

- Beck AT. Cognitive Therapy and the Emotional Disorders. New York: International Universities Press; 1976.

- Bandura A. Social Learning Theory. Englewood Cliffs, NJ: Prentice Hall; 1977.

**Trauma-Informed Care (cross-cutting)**

- Substance Abuse and Mental Health Services Administration (SAMHSA). SAMHSA's Concept of Trauma and Guidance for a Trauma-Informed Approach. HHS Publication No. (SMA) 14-4884. Rockville, MD: SAMHSA; 2014.

- van der Kolk BA. The Body Keeps the Score: Brain, Mind, and Body in the Healing of Trauma. New York: Viking; 2014.

**Implementation Science (cross-cutting)**

- Michie S, van Stralen MM, West R. The behaviour change wheel: A new method for characterising and designing behaviour change interventions. Implementation Science. 2011;6:42.

- Atkins L, Francis J, Islam R, et al. A guide to using the Theoretical Domains Framework of behaviour change to investigate implementation problems. Implementation Science. 2017;12:77.
