# Supplementary material for: Verbal de-escalation techniques for the prevention and management of workplace violence in healthcare: a systematic review
Source: Front Psychiatry. 2026 Oct 14;17:1819502. doi: 10.3389/fpsyt.2026.1819502 (PMC13713309; doi:10.3389/fpsyt.2026.1819502)
Supplement: Supplementary file 4 [file Table1.docx]

**Supplementary Table 1.** Characteristics of included studies examining verbal de-escalation techniques in healthcare settings, including MMAT quality appraisal ratings.

**Table 1.** Characteristics of the included studies.

| **Study ID** | **Country** | **Target Population & Sample Size** | **Main Verbal De-Escalation Technique(s)** | **Study Type** | **Study Duration & Data Collection** | **Measurement Parameters** | **MMAT Score**  (Mixed Methods Appraisal Tool) |
| --- | --- | --- | --- | --- | --- | --- | --- |
| Baig 2018 (37) | Pakistan | HCPs in Emergency, Gynecology & Obstetrics, Medicine & Allied and Surgery (n = 141) | Training on prevention, de-escalation, and management of verbal and non-verbal aggression; focus on practical skills to handle patient aggression | Quasi-experimental | Four sessions lasting 4 hours each, covering four different themes with follow-up 4 months after participating in the simulation | Structured questionnaire to collect self-reports on frequency, types, and reasons for violence experienced, and the Confidence in Coping with Patient Aggression (CCPAI) | Moderate |
| Baldacara 2018 (38) | Brazil | Patients with psychomotor agitation; HCPs trained in management (n not specified) | Verbal de-escalation techniques: hierarchical non-pharmacological management starting with verbal approaches, including calming communication, attentive listening, and structured patient redirection | Guideline development (systematic review + expert consensus) | Articles published 1997–2017; systematic review of 104 included studies; recommendations synthesized by a Task Force of 11 psychiatrists | Evidence levels graded using Oxford Centre for Evidence-based Medicine system; qualitative assessment of guideline recommendations | N/A |
| Brenig 2023 (39) | Germany | Mental health staff in forensic psychiatric settings (n = 8–112 across 5 studies) | Staff training in verbal de-escalation techniques: attentive listening, calm verbal communication, limit-setting, collaborative approach; hierarchical and integrated into broader staff training programs | Systematic review of non-randomized studies | Studies published 2002–2021; included 5 studies (4 before-after, 1 post-test-only); duration varied by individual training programs | Staff-reported measures: confidence, perceived safety, perceived aggressive behaviour; objective outcomes (only 2 studies): rates/severity of violent incidents, injuries, use of restraints | N/A |
| Cahill 2008 (40) | USA | Emergency department nurses (n = 12 in ED; total experimental group n = 56; control group n = 9) | Verbal de-escalation via communication techniques: empathetic tone and pace, attentive listening, limit-setting, structured communication strategies, collaborative approach; integrated into ACT-SMART training | Quasi-experimental, pretest-posttest with control group | Single 8-hr educational workshop (ACT-SMART); pre- and post-training questionnaires administered | Staff-reported: confidence in managing aggression, attitudes toward aggressive patients, perceived incidence of aggression | Moderate |
| Cowin 2003 (41) | Australia | Mental health unit (MHU) and emergency department (ED) nurses (MHU n = 21–19; ED n = 33–30 across pre/post survey) | Verbal de-escalation: limit-setting, empathetic communication, attentive listening, collaborative approach reinforced via poster and in-service discussions | Quasi-experimental, pre-post survey | 3-month intervention period; pre- and post-intervention survey; in-service education sessions and poster display | Staff-reported: knowledge and awareness of de-escalation techniques via 11-item survey (Likert-type scale) | Moderate |
| Crawford 2020 (42) | UK | Children’s nurses (sample size not specified) | Verbal de-escalation: limit-setting, empathetic communication, attentive listening, collaborative discussion with families; proactive identification of high-risk situations | Educational article / descriptive guidance | N/A – narrative discussion and time-out activities | N/A – learning outcomes assessed via reflective exercises and discussion prompts | N/A |
| Dombrowsky 2012 (43) | USA | Healthcare staff (sample size not specified; survey included 950 respondents) | Verbal de-escalation: limit-setting, controlling emotions, rapid identification of abuse, taking control of the situation, post-incident follow-up (ICE TEA framework) | Narrative / descriptive guidance | N/A – literature review and guidance | N/A – not measured quantitatively; includes survey findings on incidence of verbal abuse | N/A |
| Duncan 2021 (44) | USA | Nurses, patient service associates, protective services officers on a medical-surgical unit (n = 75) | Multidisciplinary verbal de-escalation via didactic instruction + standardized patient simulation + structured debrief; emphasis on empathy, role clarity, communication, and teamwork | Quasi-experimental, simulation-based training | Pre-test survey and a didactic session with facilitated discussion on techniques (30 min), followed by a 10-minute session using techniques and concepts taught during the didactic session with a 50-minute debrief afterwards. A post-survey was also included | Likert-based scale to evaluate confidence, simulation relevance to clinical practice, likelihood to apply knowledge learned and realism, Management of Aggression and Violence Attitude Scale (MAVAS), an open-ended questionnaire to capture reflections about the simulation | Moderate |
| Georgadarellis 2023 (45) | USA | Medical professionals caring for acutely agitated pediatric patients (ED-focused; sample not specified) | 10-domain verbal de-escalation framework: respect personal space, avoid provocation, establish verbal contact, be concise, identify wants/feelings, listen closely, agree or agree to disagree, set clear limits, offer choices/optimism, debrief patient & staff; emphasizes nonpharmacologic interventions before pharmacologic or restraint use; multidisciplinary approach | Narrative review / CME guidance | N/A (review article) | N/A (educational objectives and best-practice recommendations) | N/A |
| Gupta 2023 (25) | USA | Hospitalists (physicians and advanced practice providers, such as NPs and PAs) (n = 37) | In-person 3-hour training with didactics and hands-on role-play; included education on WPV, 5-phase assault cycle, patient behaviour-specific interventions; emphasized structured verbal de-escalation strategies and self-protection skills | Pilot interventional study (pre-post design) | April 2019 – Dec 2020; pre- and post-training surveys at immediate, 3, 6, and 12 months | Prevalence of workplace violence (verbal, racial, physical, sexual); self-reported confidence in coping with patient aggression (validated 10-item scale, score 10–110) | Moderate |
| Haefner 2021 (20) | USA | Psychiatric nurses on a 37-bed inpatient psychiatric unit (n=31); all patients admitted 2 months pre- and post-intervention (pre n=388, post n=342) | TeamSTEPPS program: 3 self-learning modules + in-class demonstrations; included posters and badge cards summarizing de-escalation steps; focused on verbal de-escalation to reduce aggressive behaviour and seclusion | Quality improvement project, quasi-experimental pre-post design | 2 months pre- and 2 months post-implementation; patient chart review and staff education evaluation | Rate of documented patient aggression and use of seclusion; demographic characteristics of patients and staff; statistical significance via Chi-square test | Moderate |
| Hallett 2015 (30) | UK | Clinical staff in mental health inpatient service (n=72) | Verbal and non-verbal communication, non-confrontational limit setting, calm talk; emphasis on early recognition, situational assessment, and staff qualities for de-escalation | Cross-sectional mixed-methods survey | July 2013 – Feb 2014; questionnaire survey with clinical vignettes | Staff definitions and conceptualizations of de-escalation, reported interventions for aggressive scenarios, beliefs about intervention efficacy; thematic analysis of qualitative responses | High |
| Homotoff 2021 (46) | USA | Healthcare professionals in general and emergency settings (sample size not specified) | Respect personal space, avoid provocation, establish verbal contact, concise communication, identify wants/feelings, active listening, agree/agree to disagree; situational awareness and self-monitoring emphasized | Narrative review / expert guidance | Not specified; descriptive discussion based on literature and case examples | Identification of aggression types, situational awareness, clinician self-awareness, workplace violence prevention practices | N/A |
| Mavandadi 2016 (47) | Canada | Mental health staff in a psychiatric hospital: nurses (n = 105), allied health (n = 22), physicians (n = 8) | Seven validated de-escalation skills (from DABS) with descriptors for best, acceptable, and least desirable practice; applied via video simulations and expert feedback to enhance staff practice | Psychometric validation study | 3-year workforce development initiative; 272 video simulations analyzed | Seven-item English Modified De-Escalating Aggressive Behaviour Scale (EMDABS); inter-rater reliability (ICC), internal consistency (Cronbach’s alpha), factor analysis | Moderate |
| Moore 2022 (48) | Australia | Clinicians in emergency nursing programs: n = 28 | VR-based conversational agent (Code Black VR) allowing clinicians to practice verbal de-escalation with a virtual patient; guided by Clinical Training Through VR Design Framework | Exploratory usability study (Virtual reality (VR) simulation) | 5 workshops (~20 min each) at simulation center; multiple VR playthroughs with post-study survey and researcher observation | System Usability Scale (SUS), thematic analysis of participant comments, in-app analytics (interactions with VR agent) | High |
| Pérez -Toribio 2022 (49) | Spain | Nurses in an acute inpatient mental health unit; 493 episodes of mechanical restraint analyzed retrospectively | Nurse-led verbal de-escalation interventions prior to mechanical restraint; aimed at calming agitation and preventing restraint, with analysis of factors associated with use | Retrospective cohort study | Patient records from Jan 2012 – Dec 2018 | Use of verbal de-escalation (yes/no) extracted from case logs; sociodemographic and clinical variables (age, sex, diagnosis, days since admission, prior restraint, pharmacological treatment, substance use) | Moderate |
| Price 2012 (50) | USA, UK, Sweden, Finland | Nursing staff in mental health services; 11 qualitative studies included in a thematic synthesis | Staff skills (characteristics of effective de-escalators, maintaining personal control, verbal & non-verbal skills); process of intervening (engaging patient, timing, safe conditions, strategies including autonomy confirming and limit-setting interventions) | Thematic synthesis literature review | Literature search of multiple databases with no date limits; 11 studies selected based on inclusion criteria | Qualitative data describing de-escalation processes; staff skills and intervention strategies | High |
| Price 2024 (51) | UK | Adult acute and forensic inpatient wards; 128 participants in qualitative inquiry, 275 professionals trained in feasibility trial | Complex co-designed intervention: de-escalation training, reflective practice models, post-incident debriefing, collaborative ward rounds, sensory modulation, structured ward practices, environmental modifications, and staff attitude/knowledge enhancement | Systematic review, qualitative inquiry, co-designed intervention, uncontrolled pre-post feasibility trial | 24-week clinical outcome data collection (8-week pre-intervention, 8-week embedding, 8-week post-intervention) | Conflict and containment rates (Patient–Staff Conflict Checklist); staff/patient attitudes (Attitudes to Containment Measures Questionnaire, Attitudes to Personality Disorder Questionnaire, Violence Prevention Climate Scale, Capabilities, Opportunities, Motivation Scale, Coercion Experience Scale, Perceived Expressed Emotion in Staff Scale) | Moderate |
| Richmond 2012 (21) | USA | Agitated patients in emergency departments; no specific sample size reported | Expert consensus statement | N/A – guidance based on expert consensus and clinical experience | N/A – focus on recommended approaches rather than quantitative outcomes | Rapid verbal de-escalation (5–10 min) using: establishing rapport, assessing agitation, empathic communication, incremental offers of medication, sympathetic and authoritative communication, one-on-one engagement, safe staff positioning, and patient-centered interventions to prevent escalation | N/A |
| Richter 2006 (52) | Germany | Mental health staff and patients in psychiatric settings; no specific sample size reported | Nonphysical conflict management: active/empathic listening, acknowledging emotions, paraphrasing, open-ended questions, I-messages, defusing verbal attacks, avoiding communication roadblocks (ordering, blaming, shaming, why-questions), leading conversation back to topic, and carefully structured concessions; emphasizes training, experience, and situational fit | Literature review / expert synthesis | N/A – narrative review | N/A – descriptive focus on techniques rather than quantitative outcomes | N/A |
| Lafta 2024 (53) | Iraq | Junior doctors in hospitals in Baghdad and Karbala; pre-training survey n=1079, trial n=190 | Two-day training covering communication skills, handling aggressive attitudes, breaking bad news, patient interaction strategies; emphasizes empathy, confidence building, and practical strategies to prevent escalation; measured by reduced incidence of violent events | Interventional randomized trial (one-arm, pre/post design) | August 2021 – July 2022; six-month pre-training and six-month post-training follow-up | Exposure to workplace violence (physical, verbal, threatening); demographic/work characteristics; pre/post training comparison | Low |
| See 2025 (54) | USA | Fourth-year medical students (n=44 curriculum students; 83 non-curriculum students) | CALMER framework: Calm, Acknowledge, Listen, Mirror, Empower, Responsibility; interactive didactic + standardized patient practice; emphasizes empathy, reflective listening, addressing concerns, and maintaining personal safety | Curriculum development & evaluation; pre/post survey and OSCE | Summer 2023; part of “Get Ready for Residency Bootcamp” | Confidence and preparedness in using verbal de-escalation (pre/post survey); OSCE performance using checklist with six CALMER skills | Moderate |
| Moir 2022 (55) | Australia | Graduate-entry nursing students (n=33) | Pre-emptive communication skills education focusing on safe and effective responses to patient aggression; emphasizes confidence-building, interpersonal communication competence, and early skill development prior to clinical exposure | Quasi-experimental pre/post design | Baseline before education module and post-completion of clinical placements in acute care and mental health | Thackrey's Confidence in Coping with Patient Aggression Instrument; Interpersonal Communication Competence Scale | Moderate |
| Power 2023 (56) | Australia | Undergraduate third-year nursing students (n=28) | Simulation-based experiential learning emphasizing role-play with standardized patients; focuses on recognition of emotional responses, reflection, and development of practical de-escalation strategies in a safe environment | Exploratory study using simulation and role-play | Immediate debrief sessions following a single simulated aggressive patient scenario | Qualitative reflections from debrief sessions | High |
| Shenai 2024 (57) | USA | Third- and fourth-year medical students on psychiatry clerkship (n=139) | Standardized patient simulation with peer observation, reflective debrief, and time-out option; focus on practicing verbal de-escalation techniques across multiple agitation phenotypes; feedback-driven iterative learning | Simulation-based SP workshop with reflective debrief; descriptive evaluation | 12 monthly sessions over 12 months; 2.5 hours per session; feedback surveys and video review of a random sample of encounters | Student self-reported achievement of learning objectives, quality ratings, thematic analysis of narrative feedback, SP observations, video review of behaviours | High |
| Adams 2017 (58) | Australia | Nurses, Assistants in Nursing, Patient Care Assistants on two adult medical wards (n=65) | Clinical education program covering assessment, planning, crisis implementation, post-incident reflection; included case studies and in-patient scenarios; focus on verbal de-escalation skills and coaching by clinical experts | Before-and-after educational intervention | Education program delivered on study wards; incident records reviewed pre- and post-intervention; 77% staff completed training | Staff knowledge, confidence, and capability in managing violent/aggressive incidents; frequency and recurrence of violent events; high-risk patient identification | Moderate |
| Celofiga 2022 (59) | Slovenia | Staff in acute psychiatric wards across 6 hospitals; 6,401 patient treatment episodes (baseline: 3,190; intervention: 3,211) | 16-hour de-escalation training covering verbal and non-verbal techniques, theoretical review, workshops, video materials, role-play; staff encouraged to integrate de-escalation into ward philosophy and use prior effective strategies for high-risk patients | Multi-center cluster randomized controlled trial | Two 5-month periods: baseline (2018) and intervention (2019); staff trained in verbal/non-verbal de-escalation | Incidence and severity of aggressive incidents (SOAS-R), number of patients with aggression, frequency and duration of physical restraints (PR), recurrent incidents | Moderate |
| Feinstein 2014 (60) | USA | Psychiatric residents and staff; ~60 participants per year (2011–2012) | Violence de-escalation training as part of 8-part curriculum, including situational awareness, risk assessment, safe environment strategies, legal considerations, and simulation exercises for practicing de-escalation | Quality and safety curriculum evaluation (pre-post with surveys and qualitative feedback) | 2 years; 8-part curriculum including didactic sessions, simulations, and video-based exercises | Participant-reported preparedness, usefulness of simulation exercise, qualitative feedback, post-simulation symptoms | High |
| Kronish 2024 (61) | USA | Pediatric residents, family medicine residents, triple-board residents; 22 participants | SP simulation of an agitated pediatric patient; verbal de-escalation practiced alongside appropriate pharmacologic selection; debrief with child/adolescent psychiatry experts focusing on reflection, team communication, and application of de-escalation techniques | Standardized patient (SP) simulation with post-case debrief | 8 sessions; each 30 min (10–15 min case, 15–20 min debrief) | Pre- and post-survey of learner confidence, qualitative feedback, facilitator observation checklist | Moderate |
| Arturén 2024 (62) | Sweden | Nursing staff (assistant nurses, registered nurses, specialist psychiatric nurse) in psychosis inpatient units (n = 11) | Interactive Approach (IA) model; structured risk scale; active listening; empathetic communication; flexible problem-solving; clear communication and boundary-setting | Qualitative descriptive study (interview study with inductive content analysis) | Semi-structured face-to-face interviews conducted September 2019 – January 2021; interviews lasted 26–52 minutes; transcribed verbatim | Semi-structured interview guide; qualitative content analysis identifying categories and subcategories of staff experiences using the IA model | High |
| Bowllan 2025 (63) | USA | Interdisciplinary psychiatric ED staff (n = 171) | High-fidelity simulation; CPI de-escalation; TeamSTEPPS communication; role clarification; structured debriefing | Quantitative non-randomized quality improvement (pre–post) | 2.5-hr simulation sessions; 5-month pre–post restraint/injury data | CCPAI; TeamSTEPPS Perception Questionnaire (T-TPQ); restraint rates; staff injury rates; educational survey; debriefing thematic analysis | Moderate |
| Duncan 2024 (64) | USA | Urban ED staff (n = 117) | Simulation-based de-escalation; didactic training; standardized patient; structured debrief; dignity framework; role clarification; trigger reduction | Quantitative non-randomized quality improvement (pre–post) | 90-min session; 12-month pre–post restraint data; 6-month follow-up survey | Modified Likert confidence surveys; 6-month retention survey; restraint rates per 1000 visits; environmental/process changes | Moderate |
| Georgadarellis 2025 (65) | USA | Pediatric ED staff (n = 61 baseline; n = 44 post-intervention) | Asynchronous visual aids, podcasts, online module, badge cards; listening, respecting space, verbal contact, avoiding provocation, identifying patient wants/feelings | Quantitative non-randomized quality improvement (pre–post) | Oct 2022 – May 2023; pre- and post-intervention electronic surveys; 3-month follow-up for behaviours | MAVAS attitude scale; knowledge questions on agitation and restraint disparities; self-reported behaviours; frequency/effectiveness of de-escalation techniques | Low |
| Phipps 2024 (66) | USA | Ambulatory clinic staff (non-physician, patient-facing; n = 332 across 4 clinical specialties) | Empathy/self-awareness training: ABC model, value listening, Four Expressions of Anger, physiological stress management, active listening, Five Whys; interactive scenarios; 2 h in-person/virtual | Quantitative non-randomized (pre–post survey) | Pre- and post-intervention questionnaires, aggregated by clinical specialty; patient satisfaction, complaints, and workplace violence cases tracked | Self-reported confidence in de-escalation and self-care (pre/post, Likert-type), participant feedback on course, patient satisfaction scores, patient complaints/grievances, workplace violence case counts | Moderate |
| Tartak 2025 (67) | USA | ED patient-facing staff (clinical and non-clinical; n = 91 training participants, 72 completed pre- and post-surveys) | Verbal de-escalation & implicit bias check: AVADE (Awareness-Vigilance-Avoidance-Defense-Escape) Workplace Violence Prevention training, trauma-informed care, implicit bias self-check, RELAXS mnemonic, simulation with standardized patients of colour, hands-on defensive skills, case-based debrief | Quantitative non-randomized (pre–post survey) | September 2022 – June 2023; 14 sessions; pre- and post-training surveys with 11 Likert-scale questions; participant role and years of experience recorded | Self-reported confidence/comfort in managing agitated patients, verbal de-escalation, bias checks, trauma-informed care, personal/team safety | Moderate |
| Thompson 2025 (68) | USA | Newly graduated nurses (n = 420 pre, 174 post) | Multimodal verbal de-escalation: didactic module, live-actor immersive simulation, team-based role-play, structured debrief focusing on early recognition, verbal strategies, therapeutic communication | Quantitative non-randomized (pre–post survey) | November 2021 – August 2022; pre-survey week 2 of hire, post-survey end of transition year; 10-item Confidence in Coping with Patient Aggression (1–10 Likert) | Self-reported confidence in handling aggression, verbal de-escalation, preparation, efficacy, personal safety, training adequacy | Low |
